# *Hyssopus officinalis* L. (Lamiaceae) Cell Culture Extract Modulates Epidermal Lipid-Related and Differentiation Markers

**DOI:** 10.3390/cells15141300

**Published:** 2026-07-21

**Authors:** Annalisa Tito, Eleonora Vardaro, Adriana De Lucia, Danila Falanga, Cristiana De Musis, Antonio Colantuono, Annamaria Cefariello, Lucia Ricci, Giuditta Vitiello, Ilaria Arra, Ritamaria Di Lorenzo, Gianfranco Diretto, Olivia Costantina Demurtas, Maria Sulli, Apostolos Pappas, Giovanni Greco, Sonia Laneri, Maria Gabriella Colucci

**Affiliations:** 1 Arterra Bioscience Spa, 80142 Naples, Italy; annalisa@arterrabio.it (A.T.); adriana@arterrabio.it (A.D.L.); danila@arterrabio.it (D.F.); cristiana@arterrabio.it (C.D.M.); antonio@arterrabio.it (A.C.); anna@arterrabio.it (A.C.); giuditta@arterrabio.it (G.V.); ilaria@arterrabio.it (I.A.); or gabriellacolucci@vitalabactive.com (M.G.C.); 2 Dipartimento di Farmacia, Università degli Studi di Napoli Federico II, 80131 Naples, Italy; eleonora.vardaro@unina.it (E.V.); lucia.ricci@unina.it (L.R.); ggreco@unina.it (G.G.); slaneri@unina.it (S.L.); 3 Italian National Agency for New Technologies, Energy, and Sustainable Development (ENEA), Green Bi Technology Laboratory, Casaccia Research Centre, 00123 Rome, Italy; gianfranco.diretto@enea.it (G.D.); olivia.demurtas@enea.it (O.C.D.); maria.sulli@enea.it (M.S.); 4 CRB S.A., CH-1070 Puidoux, Switzerland; apostolos.pappas-ext@intercos.com; 5 Vitalab S.r.l., 80142 Naples, Italy

**Keywords:** *Hyssopus officinalis*, plant cell culture, β-glucocerebrosidase, serine palmitoyltransferase, involucrin, filaggrin, ceramides, skin barrier, transepidermal water loss, cosmetic biotechnology

## Abstract

**Highlights:**

**What are the main findings?**

- *Hyssopus officinalis* cell cultures grown in the dark accumulated markedly higher levels of rosmarinic acid, hydroxytyrosol glycosides and flavonoids compared with the light-grown cultures and intact plants.
- In vitro and ex vivo models demonstrated that HoEx significantly enhanced β glucocerebrosidase activity, ceramide production, and the expression of key enzymes involved in ceramide biosynthesis (β glucocerebrosidase, serine palmitoyltransferase) as well as structural proteins (involucrin, filaggrin). In a clinical study, HoEx also increased β glucocerebrosidase activity and reduced transepidermal water loss in mechanically stressed skin.

**What is the implication of the main finding?**

- HoEx may support epidermal barrier-related functions by modulating endogenous ceramide-associated pathways and the expression of key structural proteins, providing a standardized source of bioactive metabolites with potential relevance for dermocosmetic applications.

**Abstract:**

The skin barrier is essential for maintaining epidermal homeostasis and preventing transepidermal water loss (TEWL). This study investigated the ability of a *Hyssopus officinalis* cell culture hydroethanolic extract to support epidermal barrier function by modulating ceramide-related enzymes and structural proteins. Hydroethanolic extracts from cell cultures grown under light and dark conditions were characterized by high-resolution mass spectrometry. Dark-grown cultures accumulated higher levels of rosmarinic acid, hydroxytyrosol glycosides, and polymethoxyflavones than light-grown cultures and intact plants. In keratinocytes, the extract from dark-grown cells (HoEx) at 0.002% *w*/*v* exhibited significant antioxidant activity (20% ROS reduction) and increased β-glucocerebrosidase (GBA) activity (25%) as well as the expression of β-glucocerebrosidase (44%), serine palmitoyltransferase (34%), involucrin (17%), and filaggrin (44%) compared with control. In human skin explants, 0.5% *w*/*w* HoEx increased ceramide content (36%) and total epidermal lipid levels (99%) relative to control. In a clinical study involving 10 volunteers with mechanically stressed skin, 0.5% *w*/*w* HoEx increased GBA activity by 30% and reduced TEWL by 15%, whereas placebo treatment led to a 55% decrease in GBA activity and a 25% increase in TEWL. Collectively, these findings indicate that HoEx modulates multiple epidermal pathways involved in lipid processing and differentiation under in vitro, ex vivo, and stressed in vivo conditions. While the study was not designed to establish mechanistic causality, the coordinated modulation of ceramide-related enzymes, structural proteins, and TEWL suggests that HoEx may support epidermal barrier function within the limits of the tested models. HoEx therefore represents a standardized source of bioactive compounds with potential relevance for dermocosmetic applications.

## 1. Introduction

The skin stratum corneum (SC) provides the primary barrier against environmental stressors and regulates transepidermal water loss (TEWL), ensuring epidermal homeostasis [1]. Its architecture depends on corneocytes embedded in a lipid matrix enriched in ceramides, cholesterol, and fatty acids [2]. Late differentiation proteins such as involucrin (INV) and filaggrin (FLG) contribute to cornified envelope formation and are widely used as markers of keratinocyte maturation [3,4,5].

Ceramides are central to skin barrier lipid organization. Their biosynthesis involves both de novo synthesis and glucosylceramide hydrolysis, regulated by serine palmitoyltransferase (SPT) and β-glucocerebrosidase (GBA) [6,7]. Modulation of these enzymatic pathways is increasingly explored as a strategy to support epidermal lipid homeostasis [8,9,10,11].

Plant-derived bioactive compounds have attracted interest for their ability to influence epidermal lipid metabolism, oxidative stress responses, and keratinocyte differentiation [12,13,14,15,16]. Enhancing endogenous lipid-processing pathways is considered a promising approach to support skin function. Plant cell culture technology provides a sustainable platform for producing standardized extracts enriched in specialized metabolites with dermatological relevance [17,18,19,20]. *Hyssopus officinalis*, a Lamiaceae species rich in rosmarinic acid and related phenolics, exhibits antioxidant and anti-inflammatory properties and remains underexplored in dermatology [21,22,23,24,25]. Its cell cultures offer controlled conditions to optimize metabolite accumulation and respond predictably to environmental cues such as light availability [25,26,27]. In this context, the evaluation of different culture conditions was used to identify the most stable and representative extract to be employed in the subsequent biochemical and ex vivo analyses.

Herein, we combined metabolomic profiling, in vitro assays, ex vivo analyses, and a clinical evaluation to investigate hydroethanolic extracts obtained from *Hyssopus officinalis* cell cultures grown under different light conditions. We focused on the modulation of key enzymes and markers involved in ceramide metabolism and epidermal differentiation, including GBA, SPT, INV, and FLG, to evaluate the potential relevance of the extract obtained from dark-grown cultures for epidermal barrier-related processes.

## 2. Materials and Methods

### 2.1. Extract Preparation from Hyssopus officinalis

#### 2.1.1. Extract Preparation from Cell Cultures

A certified hyssop plant (*Hyssopus officinalis*) was obtained from a local nursery (“Vivaio L’elce”, Bagno a Ripoli, Italy). Leaves were surface sterilized with 70% (*v*/*v*) ethanol (Sigma Aldrich, St. Louis, MO, USA), followed by treatment with 1% bleach supplemented with Tween 20 (Sigma Aldrich, St. Louis, MO, USA), and finally rinsed with sterile distilled water. The sterilized leaves were then cut into 0.5–1.0 cm segments and cultured on solidified Gamborg B5 medium (Merck, Darmstadt, Germany) containing 500 mg/L myo-inositol, 30 g/L sucrose, phytohormones, and 8 g/L phytoagar. Explants were subcultured every four weeks onto fresh medium for three months. Once a compact and friable callus had formed, cells were transferred to liquid Gamborg B5 medium supplemented with 500 mg/L myo-inositol, 30 g/L sucrose, 1 mg/L adenine, and phytohormones.

The suspensions were shaken on a rotary shaker at 27 °C under either a 16 h photoperiod or complete darkness, generating two distinct culture conditions. Once the liquid suspension cultures reached at least 160 g/L, cells from each condition were collected separately and processed to obtain two hydroethanolic extracts: one derived from light-grown cultures (HoEx-L) and one from dark-grown cultures (HoEx-D). Both extracts were prepared following the procedure described by Ceccacci et al. [18], with slight modifications. Briefly, 2000 mL of ethanol/water (90/10, *v*/*v*) was added to 500 g of cells. The mixture was homogenized for 3 min at 1500 rpm and then for 6 min at 3800 rpm using a Grindomix GM300 knifemill (Retsch GmbH, Haan, Germany). The resulting suspension was stirred at 400 rpm for 2 h at 25 °C, protected from light, and subsequently centrifuged at 6300 rpm for 10 min at 4 °C. The supernatant was collected, filtered, and concentrated five-fold under vacuum using a rotary evaporator (IKA RV8, IKA-Werke GmbH & Co., Staufen, Germany) set to 25 °C. Finally, the pH was adjusted to 7.0 ± 0.5 by adding two volumes of 3× PBS, and the mixture was freeze-dried to obtain a fine powder.

As will be shown, the extract obtained from dark-grown cell cultures (HoEx-D) consistently displayed the highest accumulation of bioactive metabolites and was therefore selected for all subsequent biological assays and for the clinical study. For clarity, in the present section, the term HoEx will hereafter refer exclusively to the dark-grown extract (HoEx-D).

#### 2.1.2. Extract Preparation from Plants

The preparation of the *Hyssopus officinalis* hydroethanolic extract from plants was carried out by adding 1400 mL of an ethanol/water (90/10, *v*/*v*) solution to 350 g of plant material obtained from four different plants. The mixture was homogenized for 3 min at 1500 rpm and then for 6 min at 3800 rpm using a Grindomix GM300 knifemill (Retsch GmbH, Haan, Germany). The resulting suspension was stirred at 400 rpm for 2 h at 25 °C, protected from light, and subsequently centrifuged at 6300 rpm for 10 min at 4 °C. The supernatant was collected, filtered, and concentrated five-fold under vacuum using a rotary evaporator (IKA RV8, IKA-Werke GmbH & Co., Staufen, Germany) set to 25 °C. Finally, the pH was adjusted to 7.0 ± 0.5 by adding two volumes of 3× PBS, and the mixture was freeze-dried to obtain a fine powder. 

### 2.2. LC-HESI-HRMS Analysis

The hydroethanolic extracts were analyzed by liquid chromatography–heated electrospray–high resolution mass spectrometry (LC-HESI-HRMS) using a Q-Exactive Hybrid Quadrupole-Orbitrap mass spectrometer (Thermo Fisher Scientific, Waltham, MA, USA). Metabolites were analyzed in three technical replicates as follows. Briefly, 10 mg of dried extract was resuspended in 1 mL of 75% methanol/0.1% (*v*/*v*) formic acid, spiked with 0.25 µg/mL formononetin (Sigma-Aldrich, St. Louis, MO, USA) as an internal standard, and extracted at room temperature by continuous agitation for 30 min in an MM400 mixer mill at 20 Hz. Samples were centrifuged at 20,000× *g* for 20 min, and 0.5 mL of the supernatant was transferred into PTFE filter vials (0.2 µm pore size) for LC-MS analysis. LC-HRMS conditions were as previously reported [28].

Targeted analysis was performed using in-house and literature data. Metabolites were identified based on accurate mass (PubChem), MS^2^ fragmentation patterns (MassBank and MetFrag), and comparison with authentic standards when available.

Untargeted analysis was carried out using Compound Discoverer v3.3 (Thermo Fisher Scientific, Waltham, MA, USA) as previously described [29]. Raw LC-HRMS data were processed to query mass spectral libraries available in ChemSpider, KEGG, PubChem, and mzCloud. Processing parameters included retention time (RT) alignment via the ChromAlign algorithm, a minimum original peak rating ≥6 in at least two samples, a mass tolerance of 5 ppm for detected features, and centroid filtering with a signal-to-noise (S/N) threshold of 1.5. Compound identification was performed using ChemSpider (formula or exact mass) and mzCloud (ddMS^2^). Peak areas were determined for preferred ions.

LC-HRMS features were further refined according to the following criteria: peak quality rating ≥7 in at least three samples, reference ion charge of ±1, group coefficient of variation (CV) ≤ 25% in at least one group (three replicates per group), annotation source with full match to predicted chemical formula, mass error (Δppm) ≤ 4, retention time between 0.8 and 28 min, and group area ≥ 1.00 × 10^6^. Compounds with MS^2^ annotation confidence > 60/100 were assigned using mzCloud.

Principal component analysis (PCA) plots and box-and-whisker plots were generated directly using Compound Discoverer v3.3 (Thermo Fisher Scientific, Waltham, MA, USA).

### 2.3. Cell Cultures and Skin Explants

Immortalized human keratinocytes (HaCaT) were purchased from Addexbio Technologies (San Diego, CA, USA), maintained in Dulbecco’s Modified Eagle Medium (DMEM; Sigma-Aldrich, St. Louis, MO, USA) and supplemented with 10% fetal bovine serum (FBS; Sigma-Aldrich, St. Louis, MO, USA) in 95% air and 5% CO_2_, in a humidified atmosphere at 37 °C.

Full-thickness skin explants, obtained from three healthy female donors aged 30–40 years undergoing breast surgery, were provided by a certified private clinical facility and processed immediately upon arrival. All donors provided written informed consent for the use of their skin tissues, in accordance with the Declaration of Helsinki. No enzymatic or mechanical dissociation was performed; tissues were used as intact ex vivo explants. Skin samples were cut using an 8 mm biopsy punch and cultured in 24-transwell plates in DMEM plus 10% FBS supplemented with 10% FBS and antibiotics (penicillin G/streptomycin, 100 U mL^−1^ and 100 μg mL^−1^; gentamicin/amphotericin B, 25 μg mL^−1^ and 250 ng mL^−1^), under air–liquid interface conditions at 37 °C in 5% CO_2_ humidified air.

### 2.4. Evaluation of Antioxidant Activity of HoEx in Keratinocytes (HaCaT)

The antioxidant activity in HaCaT cells was evaluated using a ROS assay based on the CM-DCFDA. Briefly, 1.8 × 10^4^ HaCaT cells were seeded into 96-well plates. After 24 h, cells were treated for 2 h with 0.0006% *w*/*v* or 0.002% *w*/*v* HoEx, or with 500 µM ascorbic acid as a positive control. Following treatment, cells were washed with PBS and incubated with the CM-DCFDA (5-(and 6)-chloromethyl-2′,7′-dichlorodihydrofluorescein diacetate; (Invitrogen Thermo Scientific, Waltham, MA, USA) at 37 °C for 45 min. After an additional PBS wash, cells were exposed to 450 µM H_2_O_2_ to induce ROS formation. Fluorescence was measured at 535 nm (excitation 485 nm) after 30 min or 1 h using a VictorNivo plate reader (PerkinElmer, Waltham, MA, USA). Results were expressed as mean ± SD from at least three independent experiments, each performed in triplicate.

### 2.5. Enzymatic Activity of β-Glucocerebrosidase (GBA) in Keratinocytes (HaCaT)

HaCaT cells were treated with 0.002% *w*/*v* HoEx for 24 h, then collected and lysed using a buffer containing 0.1 M citrate/0.2 M phosphate (pH 5.2), 0.25% sodium taurocholate, and 0.1% Triton X-100. Total protein content in each sample was quantified using the Bradford assay, and 25 µg of total protein were incubated for 2 h with 6 mM of the fluorescent GBA substrate (4-methylumbelliferyl β-D-glucopyranoside). The reaction was stopped by adding a glycine/NaOH solution (pH 10.7), and the fluorescent product was measured at ex 365 nm/em 448 nm using a VictorNivo plate reader (PerkinElmer, Waltham, MA, USA).

### 2.6. Enzymatic Activity of β-Glucocerebrosidase (GBA) in Skin Explants

Skin punches were cultured in DMEM supplemented with 10% FBS and treated under submerged conditions for 48 h with 0.002% *w*/*v* HoEx or 1 µM retinoic acid used as a comparator. After incubation, punches were treated with Dispase II (Thermo Scientific, Waltham, MA, USA) to detach the epidermis. Keratinocytes isolated from the epidermis were lysed using a buffer containing 0.1 M citrate/0.2 M phosphate (pH 5.2), 0.25% sodium taurocholate, and 0.1% Triton X-100. A total of 20 µg of protein was used to measure GBA activity according to the previously described protocol.

### 2.7. HoEx-Induced Modulation of Epidermal Barrier Gene Expression in Keratinocytes

To evaluate the effect of HoEx on epidermal barrier-related genes, the expression of human GBA, SPT, INV and FLG was measured in treated keratinocytes by semi-quantitative PCR. Keratinocytes (HaCaT) were seeded in 6-well plates at a density of 1.5 × 10^4^ cells per well. The cells were incubated for 6 h with 0.002% *w*/*v* HoEx or with 1 µM retinoic acid used as a comparator and then collected for total RNA extraction. All experiments were performed under standard proliferative conditions, without induction of keratinocyte differentiation. Gene expression values therefore reflect basal transcriptional modulation in undifferentiated HaCaT cells. Total RNA was isolated using the GenElute Mammalian Total RNA Purification Kit (Sigma-Aldrich, Milan, Italy) according to the manufacturer’s instructions. cDNA synthesis and PCR amplification were performed using gene-specific primers. The amplification band corresponding to each target gene was normalized to the 18S rRNA reference gene using the pair of universal primers 18S primer/competimer (Invitrogen-Thermo Scientific, Waltham, MA, USA) as internal standards. The PCR products were separated on 1.5% agarose gel, viewed using the iBright instrument (Invitrogen-Thermo Scientific, Waltham, MA, USA).

The following genes were analyzed. GBA-Fw: AGTTGCACAACTTCAGC GBA-Rv: GTCCAGGTACCAATGTAC; SPT-Fw: TACGAGGCTCCTGCTTACCA SPT-Rv: ATAGCACTGGCTATGGTGGC; INV-Fw: ATGTCCCAGCAACACACA INV-Rv: TCTGGGAGCTCCAACAGT; FLG-Fw: AGAGCTGAAGGAACTTCTGG FLG-Rv: GTGTCATAGGCTTCATCC.

### 2.8. Ceramides and Epidermal Lipids Quantification in Skin Explants

Immunohistochemistry (IHC) of ceramides and staining of epidermal lipids were performed on skin sections. Three skin biopsies, derived from each donor, were treated with a cosmetic formulation containing 0.5% *w*/*w* HoEx or placebo formulation for 72 h. After each experiment, skin punches were fixed in 4% paraformaldehyde (Merck KGaA, Darmstadt, Germany), incubated sequentially in 15% and then 30% sucrose (Merck KGaA, Darmstadt, Germany), and finally embedded in OCT freezing compound (Kaltek Srl, Saonara, Italy). Sections of 5–20 µm thickness were obtained using a CM1520 cryostat (Leica Microsystems, Wetzlar, Germany). All staining and quantification procedures were performed in triplicate for each biopsy.

Cryosections were blocked at room temperature for 1 h using a solution containing 6% BSA (Euroclone S.p.A., Pero, Italy), 5% goat serum (Merck KGaA, Darmstadt, Germany), 20 mM MgCl_2_ (Merck KGaA, Darmstadt, Germany), and 0.2% Tween-20 (Merck KGaA, Darmstadt, Germany).

For ceramide IHC, slides were processed according to the VECTASTAIN Universal Quick kit (Vector Laboratories, Newark, CA, USA), followed by incubation with anti-ceramide primary antibodies (Enzo Biochem Inc., Farmingdale, NY, USA) overnight at 4 °C. Slides were then developed using the Vector VIP kit (Vector Laboratories Inc., Newark, CA, USA). For epidermal lipid staining, slides were permeabilized with 0.5% digitonin and subsequently incubated with LipidTOX (Invitrogen, Waltham, MA, USA). The nuclei were stained with DAPI (40, 6-5 diamidino-2-phenylindole) 1 g/mL in PBS for 10 min.

For both procedures, sections were mounted with Mowiol (Merck KGaA, Darmstadt, Germany). Imaging was performed using a Leica fluorescence microscope (Leica Microsystems, Wetzlar, Germany), and images were processed using ImageJ software 1.54f (National Insititutes of Health, Bethesda, MD, USA). Values derived from ImageJ analysis are expressed as percentages relative to the placebo, set as 100%.

### 2.9. Clinical Evaluation of β-Glucocerebrosidase (GBA) Activity and TEWL

The effect of 0.5% *w*/*w* HoEx on β-glucocerebrosidase (GBA) activity in vivo was assessed in a clinical study involving 10 healthy female volunteers with Fitzpatrick skin phototypes I–IV and clinically dry skin. The study was conducted in accordance with the principles of the Declaration of Helsinki and Good Clinical Practice guidelines. All participants provided written informed consent prior to enrollment.

To induce a transient impairment of the skin barrier, both legs were subjected to standardized mechanical stress through professional waxing. Immediately afterward, the right leg was treated with a cosmetic formulation containing 0.5% *w*/*w* HoEx, while the contralateral leg received a placebo formulation, following a randomized intra-individual design. The full ingredient list of both formulations is reported in Appendix A (Table A1).

**Figure 1:**
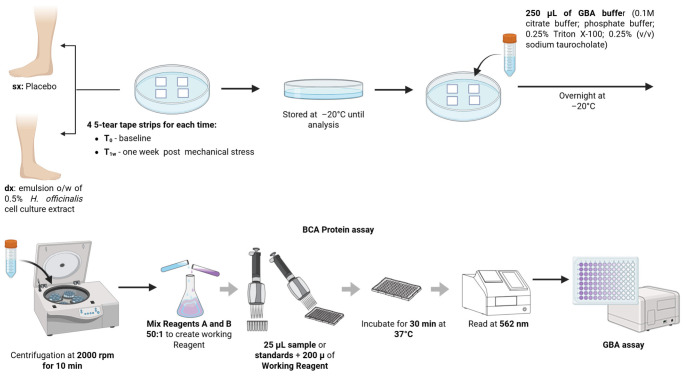
Schematic representation of the analytical workflow for β-glucocerebrosidase (GBA) in vivo assessment. Corneofix^®^ CF 20 strips were collected at baseline, after waxing, and during treatment. Corneocytes were stored at −20 °C, lysed in citrate–phosphate buffer, and total protein content was quantified by BCA assay. GBA activity was measured using a fluorometric assay.

GBA activity was evaluated at baseline (T_0_), prior to mechanical stress induction, and after one week of daily application of the respective formulations (T_1w_). At each time point, transepidermal water loss (TEWL) was measured on the treated areas using a Tewameter^®^ TM Hex probe (Courage + Khazaka Electronic GmbH, Cologne, Germany). Subsequently, tape stripping was performed on the same areas using Corneofix^®^ CF 20 strips (Courage + Khazaka Electronic GmbH, Cologne, Germany) to collect corneocytes from the stratum corneum, as shown in Figure 1. For each sampling, 4–5 consecutive tape strips were collected and immediately stored at −20 °C until analysis.

For protein extraction, the strips were incubated overnight at −20 °C in 250 µL of lysis buffer containing citrate/phosphate buffer, 0.25% Triton X-100, and 0.25% (*v*/*v*) sodium taurocholate. Samples were then centrifuged at 2000 rpm for 10 min, and the resulting supernatants were collected.

Total protein content was quantified using a BCA Protein Assay Kit (Thermo Fisher Scientific, Waltham, MA, USA), according to the manufacturer’s instructions. Based on protein quantification, equal amounts of protein (25 µg), as required by the assay protocol, were incubated with the specific fluorogenic substrate, and fluorescence was measured at 562 nm using a VictorNivo microplate reader (PerkinElmer, Waltham, MA, USA).

GBA activity was expressed relative to baseline values, using assay conditions comparable to those applied in the in vitro and ex vivo analyses.

### 2.10. Statistical Analysis

Data are presented as mean ± standard deviation (SD). For the clinical intra-individual study design, normality of the paired differences between HoEx-treated and placebo-treated sites was assessed using the Shapiro–Wilk test and confirmed by visual inspection of Q–Q plots (see Supplementary Figure S3). The distribution did not significantly deviate from normality (*p* = 0.89), supporting the use of parametric analyses. Paired Student’s *t*-tests and two-way repeated-measures ANOVA were used for comparisons versus T_0_ and placebo, respectively.

For in vitro and ex vivo experiments, differences between groups were analyzed using unpaired two-tailed Student’s *t*-tests. Statistical significance was set at *p* < 0.05. All analyses were performed using GraphPad Prism (version 10.2.2). 

## 3. Results

### 3.1. Metabolomic Characterization of Hyssopus Officinalis Cell Culture Extracts Grown Under Dark (HoEx-D) or Light (HoEx-L) Conditions

Comparative chemical profiling of hydroethanolic extracts obtained from *Hyssopus officinalis* cell cultures grown under dark (HoEx-D) or light (HoEx-L) conditions was performed using targeted and untargeted LC-HESI-HRMS metabolomics analyses. A hydroethanolic extract from adult plants was also included to compare plant- and cell-derived metabolite profiles.

**Figure 2:**
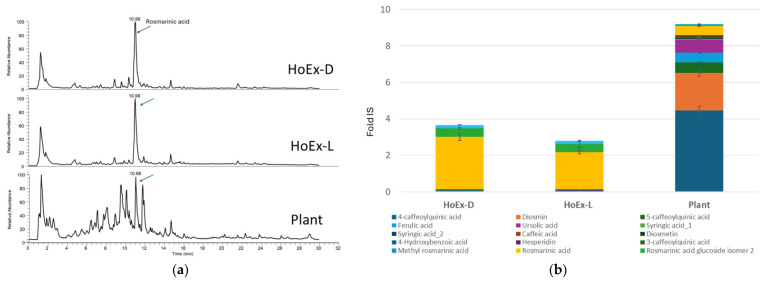
Metabolomic analysis of *Hyssopus officinalis* cell cultures grown under dark and light conditions and in adult plants. (**a**) Total ion current (TIC) chromatograms of hydroethanolic extracts analyzed by LC-HESI-HRMS. (**b**) Main accumulated polyphenols. Data are expressed as fold relative to the internal standard (IS, formononetin) and represent the mean of three independent replicates (see Table S1).

A targeted metabolomics approach according to literature [30] was used to analyze both cell cultures and plant tissues. Rosmarinic acid and its glucosides were among the most abundant metabolites in cell cultures, particularly in HoEx-D (Figure 2 and Table A2).

To further investigate the metabolite composition of cell cultures and adult plant tissues (roots, stems, and leaves), an untargeted metabolomics workflow was performed using Compound Discoverer v3.3 (Thermo Fisher Scientific, Waltham, MA, USA), including feature deconvolution, alignment, and annotation of LC-HESI-HRMS raw data (Figure S2). Low-quality and background signals were removed through blank subtraction, peak-quality filters, and accurate-mass constraints (Δppm < 4; see Section 2).

**Figure 3:**
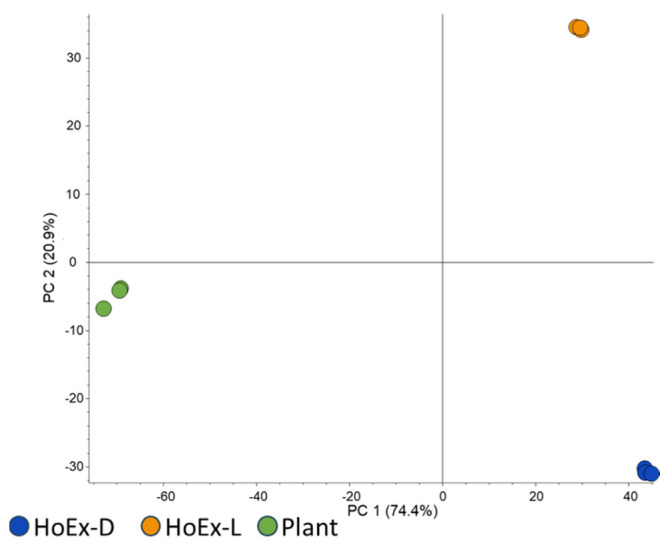
Principal component analysis (PCA) of metabolite profiles from *Hyssopus officinalis* plant tissue and cell cultures grown under dark (HoEx-D) or light (HoEx-L) conditions. PC1 and PC2 explained 74.4% and 20.9% of total variance, respectively.

Principal component analysis (PCA) revealed a clear remodulation of the metabolome in cell cultures compared to plant tissues (Figure 3). PC1 accounted for most of the variance, separating plant material from cultured cells, whereas PC2 explained a smaller portion of the variance and distinguished HoEx-L from HoEx-D, indicating that culture conditions induce subtle but consistent metabolic differences.

Using group area ratio thresholds (<0.5 or >2) and an adjusted *p*-value < 0.05, nearly 180 differentially abundant (DA) compounds were identified, over 80% of which were tentatively annotated. These metabolites included hydroxycinnamic and phenolic acids, flavonoids, amino acid derivatives, terpenes, and glycosides (Table S1).

Comparison of both culture conditions with plant extracts revealed 90 up-accumulated and 85 down-accumulated compounds. The phenylpropanoid derivative (7S,8S)-syringoyl glycerol-9-O-(6-O-cinnamoyl)-β-D-glucopyranoside—abundant in plant extracts—was largely undetectable in cell cultures. Glucuronides were also markedly reduced in cell cultures, except for ferulic acid 4-O-glucuronide, which increased 7.8-fold in HoEx-L and 75-fold in HoEx-D. Several phenolic glycosides, including caffeic acid 3-glucoside and salicylic acid glucoside, showed a significant increase (4.4- to 29.1-fold).

**Figure 4:**
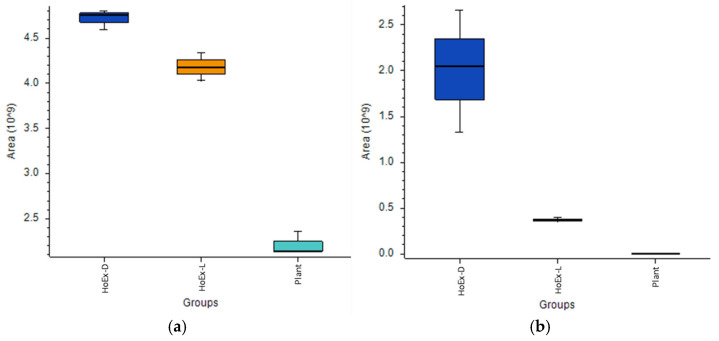
Box-and-whisker plots showing the levels of phenylethanoid-derived metabolites in *Hyssopus officinalis* extracts. (**a**) Hydroxytyrosol glucoside. (**b**) Verbascoside-like compound (*m*/*z* 623.1971) see Figure S2. Data refer to extracts from cell cultures grown under dark (blue) or light (orange) conditions and from whole-plant tissue (light blue).

Two phenylethanoid glycosides of cosmetic interest—hydroxytyrosol glucoside and a verbascoside-like compound at *m*/*z* 623.1971—also accumulated strongly in cell cultures. The latter, annotated by MS^2^ as 2-(3,4-dihydroxyphenyl)ethyl 3-O-(6-deoxy-β-L-mannopyranosyl)-6-O-[(2E)-3-(3,4-dihydroxyphenyl)-2-propenoyl]-β-D-glucopyranoside, showed a dramatic increase in HoEx-D (Figure 4), reaching levels more than 1000-fold higher than in the plant extract and 5.5-fold higher than in HoEx-L.

The untargeted analysis also revealed numerous flavonoids, including various polymethoxyflavones (PMFs) that accumulated more strongly in cell cultures. These included 5-hydroxy-3,7-dimethoxy-3′,4′-methylenedioxyflavone and 2-(3,4-dimethoxyphenyl)-5-hydroxy-3,7,8-trimethoxy-4H-chromen-4-one, with increases up to 40.2-fold compared to plant extracts.

Finally, several hydroxycinnamic acids and derivatives showed pronounced differences, particularly 4-caffeoylshikimic acid, which increased 461.8-fold in HoEx-D and 162.4-fold in HoEx-L. Similarly, 1-O-feruloyl-β-D-glucopyranose accumulated more strongly in HoEx-D (12.7-fold vs. plant extract). Consistent with the targeted analysis, rosmarinic acid was the most abundant compound in cell cultures (see Area Max. detected in Table A2), together with its methylated and glucosylated derivatives, showing on average a 5.4-fold increase—particularly under dark growth conditions.

Taken together, targeted and untargeted metabolomic analyses showed that the hydroethanolic extract obtained from dark-grown cell cultures (HoEx-D) consistently presented a higher accumulation of bioactive metabolites—including rosmarinic acid, phenolic glycosides, phenylethanoid glycosides, and hydroxycinnamic acid derivatives—compared with the extract from light-grown cultures (HoEx-L). Consequently, only the HoEx-D extract was selected for all subsequent biological assays and the clinical study and hereinafter simply referred to as HoEx.

### 3.2. Antioxidant Activity of HoEx in Keratinocytes

**Figure 5:**
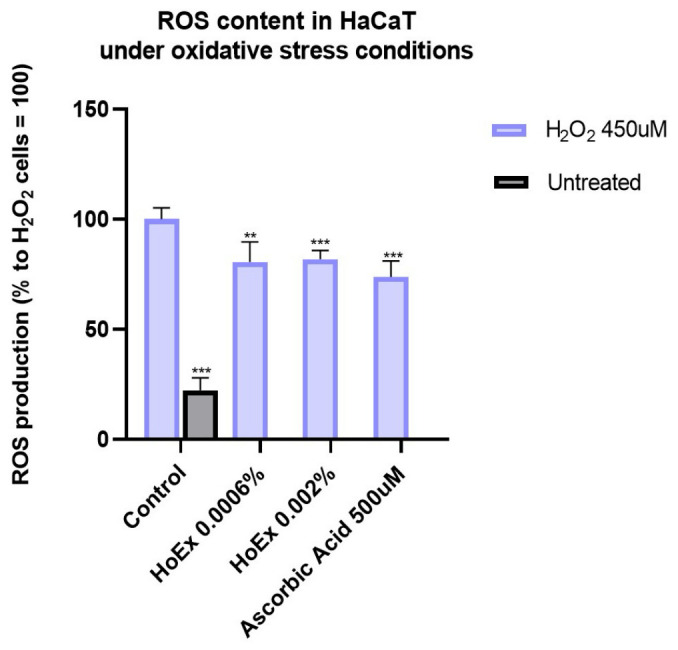
Reduction of cytosolic ROS in HaCaT cells treated with 0.0006% *w*/*v* or 0.002% *w*/*v* HoEx and exposed to H_2_O_2_-induced oxidative stress. Cytosolic ROS were measured after 30 min of stimulation with 450 µM H_2_O_2_. Ascorbic acid (500 μM) was used as a positive control. Values are expressed as percentages relative to untreated stressed cells, set as 100%. Data represent means ± SD of three independent biological replicates, each performed in triplicate. ** *p* < 0.01; *** *p* < 0.001.

Given the high rosmarinic acid content of HoEx, its antioxidant activity was evaluated in keratinocytes subjected to oxidative stress induced by H_2_O_2_. As shown in Figure 5, both 0.0006% *w*/*v* and 0.002% *w*/*v* HoEx reduced intracellular reactive oxygen species (ROS) levels by approximately 20%, with statistically significant differences compared to untreated stressed cells. Ascorbic acid, a well-known antioxidant molecule, was used as a positive control.

### 3.3. GBA Activity in Keratinocytes and Ex Vivo Skin Explants

**Figure 6:**
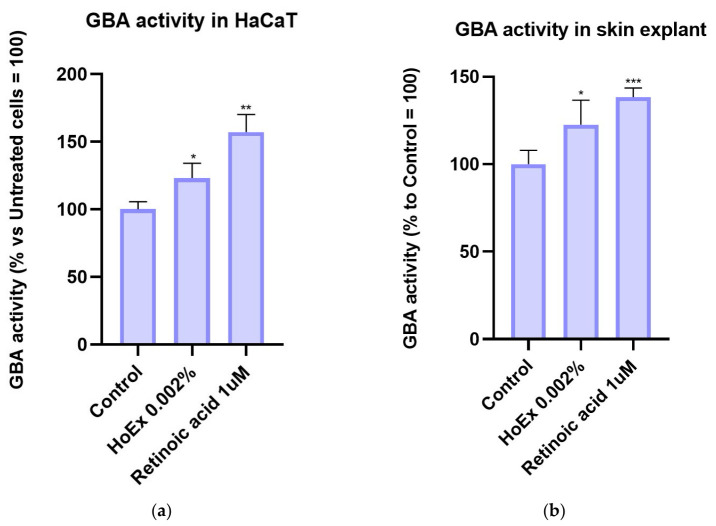
Increase in GBA activity induced by 0.002% *w*/*v* HoEx in HaCaT cells (**a**) and skin explants (**b**). GBA activity was measured in HaCaT cells treated for 24 h or in epidermal lysates from skin explants treated for 48 h. Values are expressed as percentages relative to untreated control, set to 100%. Retinoic acid (1 µM) was included as a comparator. The graphs show means ± SD from three independent biological replicates, each performed in triplicate. * *p* < 0.05; ** *p* < 0.01; *** *p* < 0.001.

The effect of HoEx on GBA activity was evaluated using systems with increasing levels of biological complexity. The first system consisted of a monolayer of keratinocytes treated with 0.002% *w*/*v* HoEx. As shown in Figure 6a, HoEx significantly increased GBA activity by 25% compared with untreated control. Retinoic acid, used as a comparator, produced a greater enhancement of GBA activity.

The second, more complex system was based on skin explants obtained from three different donors undergoing surgical procedures. Skin punches were collected and treated with 0.002% *w*/*v* HoEx for 48 h. After treatment, the whole epidermis was separated from the dermis, lysed, and GBA activity was measured in the extracted proteins. As reported in Figure 6b, HoEx treatment resulted in a statistically significant 22% increase in GBA activity compared with untreated control, indicating that HoEx is effective also in a complex tissue environment. Retinoic acid was included as a comparator with also in this ex vivo assay and showed a marked increase in GBA activity.

### 3.4. Gene Expression Analysis in Keratinocytes Treated with HoEx

**Figure 7:**
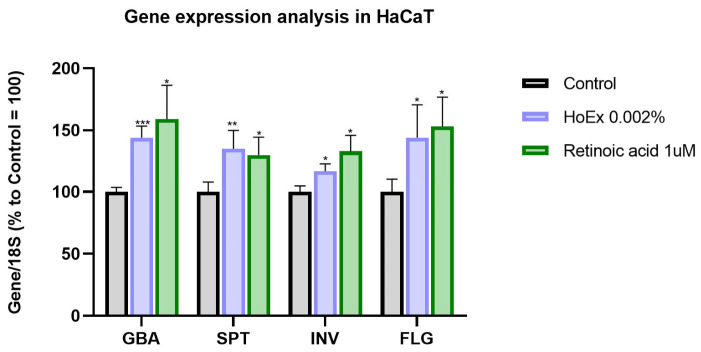
Increase in GBA, SPT, INV, and FLG gene expression in HaCaT cells treated with HoEx for 30 h. Gene expression was assessed by semiquantitative RT-PCR, and values are expressed as percentages relative to untreated control, set to 100%. Retinoic acid (1 µM) was included as a comparator. The graphs report means ± SD from three independent biological replicates, each performed in triplicate. * *p* < 0.05; ** *p* < 0.01; *** *p* < 0.001.

To assess whether HoEx influences epidermal barrier regulation, the expression of key skin-barrier-related genes was analyzed in keratinocytes treated with 0.002% *w*/*v* HoEx, using retinoic acid as a comparator. As shown in Figure 7, HoEx significantly upregulated the expression of GBA (44%), SPT (34%), INV (17%), and FLG (44%) compared with untreated control. Retinoic acid also increased the expression of these genes under the same experimental conditions. These changes were observed in undifferentiated HaCaT cells maintained under standard proliferative conditions; the control level represents basal gene expression.

### 3.5. Effect of HoEx on Ceramide Production and Total Epidermal Lipid Content in Ex Vivo Skin Explants

**Figure 8:**
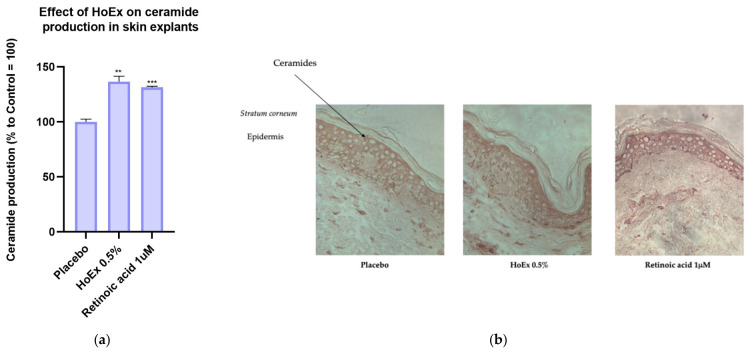
Increase in ceramides induced by 0.5% *w*/*w* HoEx in skin explants. Ceramide levels were quantified in cryosections obtained from skin explants treated for 72 h. Values derived from ImageJ analysis are expressed as percentages relative to the placebo, set as 100% (**a**). Means ± SD from three independent biological experiments, each performed in triplicate, are shown. ** *p* < 0.01; *** *p* < 0.001. Representative images of skin explants treated with 0.5% *w*/*w* HoEx or 1 µM retinoic acid, used as a comparator, are shown in panel (**b**).

The effect of HoEx on epidermal ceramides was evaluated in ex vivo skin explants treated with a cosmetic formulation containing 0.5% *w*/*w* HoEx or placebo. At the end of the treatment period, samples were processed by immunohistochemistry using a ceramide-specific antibody. As shown in Figure 8, HoEx significantly increased epidermal ceramide immunostaining signal by 36% compared with placebo-treated samples. This effect was comparable to that observed with retinoic acid (1 μM), used as a comparator. No mechanistic analyses were performed to determine the metabolic origin of the detected ceramides.

**Figure 9:**
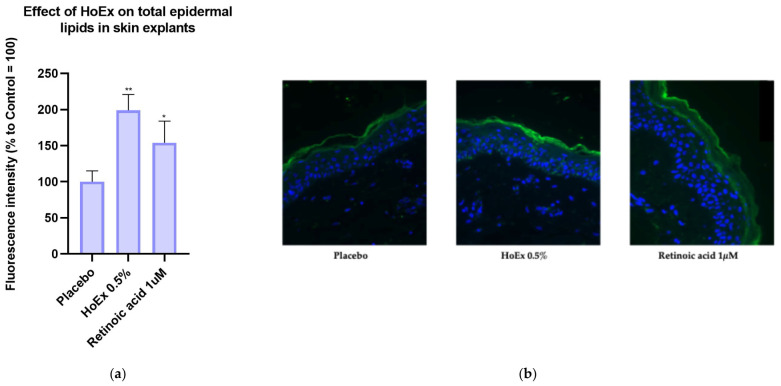
Increase in total epidermal lipids induced by 0.5% *w*/*w* HoEx in skin explants. Epidermal lipid content was quantified in cryosections obtained from skin explants treated for 72 h. Values derived from ImageJ analysis are expressed as percentages relative to the placebo, set as 100% (**a**). Means ± SD from three independent biological experiments, each performed in triplicate, are shown. * *p* < 0.05; ** *p* < 0.01. Representative images of skin explants treated with 0.5% *w*/*w* HoEx or 1 µM retinoic acid, used as a comparator, are shown in panel (**b**) in which epidermal lipids were stained in green using LipidTox dye and the nuclei (blue) were stained with 4’,6-diamidine-2-phenylindole (Dapi).

Furthermore, total epidermal lipid content was evaluated in the same skin explants, and the results are presented in Figure 9. HoEx 0.5% *w*/*w* significantly increased epidermal lipid staining intensity by 99% compared with placebo, a result that reflects total lipid staining in epidermal sections. This effect was even greater than that induced by retinoic acid, used as a comparator.

### 3.6. Clinical Evaluation of Skin GBA Activity and TEWL Under Acute Mechanical Stress

The effect of 0.5% *w*/*w* HoEx on skin GBA activity and TEWL under acute mechanical stress conditions was evaluated in a cohort of 10 volunteers. Baseline values (T_0_) were obtained on intact, non-stressed skin. After the induction of acute mechanical stress, both 0.5% *w*/*w* HoEx and the placebo formulations were applied to compromised skin areas, and GBA activity was evaluated after one week of daily treatment (T_1w_) on stratum corneum collected by tape strips (Corneofix^®^ CF 20, C+K electronic GmbH, Köln, Germany).

**Figure 10:**
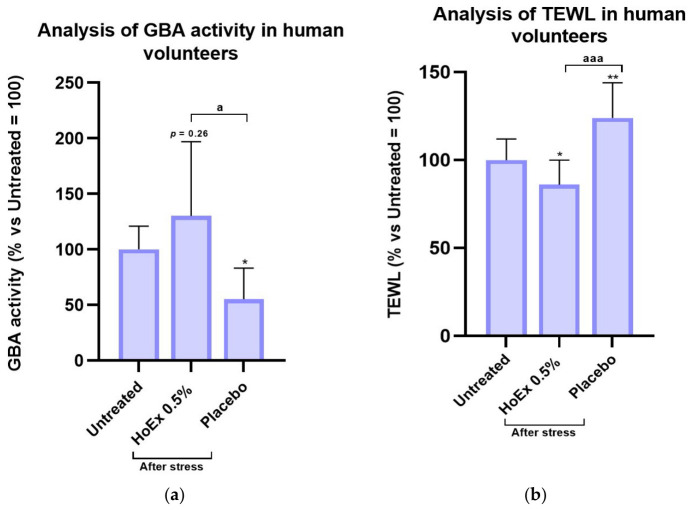
GBA activity and TEWL measurements under sustained mechanical stress. (**a**) GBA activity in tape strips collected after treatment with 0.5% *w*/*w* HoEx or placebo, expressed as percentage relative to placebo-treated sites (set to 100%). (**b**) TEWL values measured on the same areas. Means ± SD from ten volunteers, each performed in ten replicates, are shown. Asterisks indicate statistically significant differences versus untreated (* *p* < 0.05, ** *p* < 0.01), whereas “a” symbols above brackets indicate statistically significant differences versus placebo (^a^ *p* < 0.05, ^aaa^ *p* < 0.001).

As shown in Figure 10a, treatment with 0.5% *w*/*w* HoEx produced a 30% increase in GBA activity relative to baseline, whereas placebo-treated sites exhibited a 55% reduction. A comparable pattern was observed for TEWL: application of 0.5% *w*/*w* HoEx led to a 15% decrease compared with baseline, while placebo sites showed a 25% increase (Figure 10b). Individual TEWL percentage changes and the statistical comparison between HoEx- and placebo-treated sites are reported in Figure S3.

These findings indicate that 0.5% *w*/*w* HoEx effectively counteracts the barrier deterioration induced by mechanical stress. Unlike the untreated baseline, which reflects the physiological condition of intact skin, both HoEx- and placebo-treated areas were evaluated under acutely impaired conditions. Within this stressed environment—characterized by reduced GBA activity and elevated TEWL—0.5% *w*/*w* HoEx not only prevented the decline observed in placebo-treated skin but also promoted a measurable recovery of enzymatic function and barrier integrity.

Throughout our clinical investigation, no adverse events, irritation, or signs of poor tolerability were observed in any of the volunteers, supporting the good dermal compatibility of the tested formulation under the conditions of use. The active ingredient safety was evaluated using human primary skin irritation test (patch test). The test product, applied undiluted under occlusive conditions onto intact back skin of 20 volunteers, showed an average irritation index of 0 at both 15 min and 24 h after patch removal. According to the adopted classification, the product can be considered non-irritant when applied on human skin.

## 4. Discussion

The skin barrier plays a central role in maintaining epidermal homeostasis and protecting the body from environmental stressors [9]. Among its lipid components, ceramides are essential for the organization of the stratum corneum matrix and for regulating water exchange across the epidermis [31,32,33]. Altered ceramide levels have been associated with impaired epidermal conditions [9], and strategies aimed at supporting endogenous ceramide metabolism are increasingly explored in dermatological research [31,32,33]. In this study, we investigated a hydroethanolic extract obtained from dark-grown *Hyssopus officinalis* cell cultures (HoEx) to evaluate its ability to modulate epidermal lipid-related and differentiation-associated processes.

Plant cell cultures offer controlled and reproducible growth conditions that can favour the accumulation of specific metabolites of dermatological relevance, reducing variability linked to environmental factors [26,27,28,34]. A key aspect to consider is that the metabolic composition of plant cell culture extracts cannot be assumed to mirror that of the adult plant. Secondary metabolite accumulation is highly species-dependent and influenced by environmental factors such as light availability, nutrient composition, and growth regulators [26,27]. Therefore, comparing extracts obtained under different culture conditions was necessary to identify the system with the most favourable and reproducible metabolite profile.

Targeted and untargeted metabolomic analyses revealed extensive metabolic remodelling in cell cultures. Several classes of phenolic compounds—including phenylethanoid glycosides, flavonoids, hydroxycinnamic acid derivatives, and particularly rosmarinic acid—were enriched under dark growth conditions, consistent with known stress-related pathways in Lamiaceae species [35,36,37,38]. These metabolites are widely described for antioxidant, anti-inflammatory, and lipid-modulating activities [23,24,25]. However, the present study was not designed to dissect the contribution of individual constituents to the observed biological effects.

HoEx increased GBA activity in both keratinocytes and ex vivo human skin explants and upregulated the expression of GBA and serine palmitoyltransferase (SPT) in keratinocytes, indicating that the extract modulates multiple components of ceramide-related pathways at the transcriptional level. In keratinocytes, HoEx also increased the expression of involucrin (INV) and filaggrin (FLG), indicating modulation of genes involved in epidermal differentiation. These transcriptional effects—upregulation of GBA, SPT, INV, and FLG—were observed in undifferentiated HaCaT cells maintained under standard proliferative conditions, confirming that HoEx modulates ceramide-associated and differentiation-related genes without inducing keratinocyte differentiation. The functional implications of these transcriptional changes were not assessed in this study. To contextualize keratinocyte responsiveness in both the in vitro and ex vivo assays, retinoic acid was included as a comparator.

While GBA is a key enzyme in ceramide metabolism, the physiological significance of its modulation is context-dependent and cannot be attributed to a specific metabolic route based on the in vitro and ex vivo data. At this stage, increased GBA expression or activity cannot be interpreted as inherently advantageous for epidermal homeostasis or barrier function, nor as indicative of barrier impairment, as such modulation may reflect either a homeostatic repair response or a compensatory adaptation to barrier perturbation [6,39].

In skin explants, HoEx increased ceramide immunostaining and total epidermal lipid staining. The antibody used for ceramide measurements recognizes multiple species (including C16 and C24) without providing information on specific ceramide class distribution, metabolic origin, or GBA-dependent glucosylceramide turnover.

Taken together, the in vitro and ex vivo findings indicate that HoEx engages multiple components of ceramide-related pathways and promotes increases in epidermal lipids and differentiation-associated markers. Although each individual readout has intrinsic interpretative limitations, their overall pattern is seemingly more consistent with a supportive context for epidermal barrier homeostasis.

In a randomized intra-individual study on mechanically stressed skin, HoEx treatment led to an increase in GBA activity together with a reduction in TEWL. No functional assessments of barrier performance were conducted. Differences between HoEx and placebo reflect the specific outcomes measured within this experimental model. Specifically, the relationship between GBA activity, ceramide metabolism, and barrier dynamics has been extensively characterized in classical acute barrier-disruption models, where lipid extraction or repeated tape stripping triggers a well-defined recovery response [3,40]. In these systems, TEWL typically normalizes within days, and GBA activity transiently increases during early repair phases. However, the clinical protocol used in this study involves sustained mechanical stress rather than acute lipid depletion, a condition in which epidermal adaptations are less comprehensively characterized may not fully correspond to the well-defined recovery kinetics described in classical acute barrier disruption models [41]. Recent work on corneocyte biology and molecular moisturization supports the notion that prolonged mechanical or environmental stress can alter proteolytic activity, lipid processing, and TEWL trajectories in ways that differ from acute disruption [2,5,42]. Within our experimental context, placebo-treated sites exhibited a marked decrease in GBA activity together with an increase in TEWL, a pattern consistent with impaired barrier status. In contrast, HoEx-treated sites showed an increase in GBA activity accompanied by a reduction in TEWL. The concomitant decrease in TEWL therefore suggests that, under sustained mechanical stress, the increase in GBA represents an adaptive or reparative response rather than a marker of barrier deterioration. A similar pattern of coordinated epidermal responses was observed in the preclinical assays, where HoEx modulated key ceramide-related enzymes and broader lipid- and differentiation-associated markers across several readouts. These data indicate that the modulation of GBA in vivo occurs within a broader framework of lipid-related and differentiation-associated processes engaged by the extract across multiple experimental systems.

The combined in vitro, ex vivo, and in vivo findings are consistent with current knowledge on epidermal lipid metabolism and differentiation. Ceramides are central regulators of stratum corneum structure and barrier function [6,32], and alterations in enzymes involved in their metabolism—including GBA and SPT—have been linked to impaired epidermal homeostasis [8,10,11,43]. The increase in GBA activity and the upregulation of GBA and SPT gene expression observed in our models are in line with previous reports describing the modulation of these regulatory mechanisms by bioactive lipids and phenolic compounds [23,25,35,42]. Stratum corneum-specific ceramide analysis will be needed to establish whether the epidermal lipid changes observed ex vivo extend to the outermost barrier layer.

Rosmarinic acid and related metabolites have been shown to modulate epidermal lipid processing and influence NHE1-dependent acidification [23,24,25], a mechanism known to affect GBA catalytic efficiency. Similarly, the upregulation of INV and FLG is consistent with the literature describing the sensitivity of differentiation markers to phenolic constituents and oxidative stress modulators [11,35,43]. While the specific contribution of individual metabolites cannot be determined in the present study, our findings extend existing evidence by demonstrating that a metabolite-rich extract from *Hyssopus officinalis* cell cultures can modulate multiple epidermal pathways under in vitro and ex vivo conditions and that this activity is achieved with excellent skin tolerability in vivo.

To our knowledge, no previous clinical study has evaluated the effects of *Hyssopus officinalis*-derived preparations on epidermal lipid-related pathways or GBA activity. The present data therefore provide preliminary in vivo evidence supporting the biological relevance of the extract under stressed conditions. The clinical investigation was conducted in a small cohort (*n* = 10), which restricts statistical power and limits generalizability. Further studies, including disease-relevant models and larger populations, will be required to clarify biological basis of HoEx activity and to explore its potential applications.

## 5. Conclusions

A hydroethanolic extract obtained from dark-grown *Hyssopus officinalis* cell cultures (HoEx) was characterized through integrated metabolomic, in vitro, ex vivo, and exploratory in vivo analyses. The extract modulated multiple epidermal pathways, including genes involved in ceramide-related lipid metabolism and keratinocyte differentiation. These molecular and ex vivo findings were paralleled by in vivo observations in a mechanically stressed skin model, where HoEx treatment was associated with increased GBA activity and reduced TEWL under the tested conditions. While these results support the biological relevance of the extract, they do not allow conclusions regarding specific mechanistic routes or functional barrier outcomes. The use of plant cell cultures provides a controlled and sustainable platform for generating standardized bioactive metabolites of potential dermatological interest. Future studies aimed at identifying the active constituents, clarifying their mechanisms of action, and validating these effects in larger and disease-relevant models will be essential to define the potential applications of HoEx in skin research.

## Data Availability

The original contributions presented in this study are included in the article/Supplementary Materials. Further inquiries can be directed to the corresponding author.

## Supplementary Materials

The following supporting information can be downloaded at: https://www.mdpi.com/article/10.3390/cells15141300/s1; Figure S1: Q–Q plot of the paired differences in TEWL percentage change [Δ%(HoEx) − Δ%(Placebo)] obtained from the intra-individual comparison of skin sites treated with 0.5% *w*/*w* HoEx or placebo (*n* = 10). The distribution of paired differences closely followed the theoretical normal distribution, with no evident departures from linearity; Figure S2. Compound detected at m/z 623.1971, identified as 2-(3,4-Dihydroxyphenyl)ethyl 3-O-(6-deoxy-β-L-mannopyranosyl)-6-O-[(2E)-3-(3,4-dihydroxyphenyl)-2-propenoyl]-β-D-glucopyranoside by match with mzCloud MS2 fragmentation pattern; Table S1: LC-HESI-MS untargeted comparative analysis of semipolar compounds of *H. officinalis* plant and cell cultures, grown in dark (HoEx-D) and light conditions (HoEx-L), extracts cv. Levels of accumulation are reported as group area under the *m*/*z* peak of the preferred ion. Ratio HoEx-D/Plant and HoEx-L/Plant column cells are colored indicating significant differentially abundant compounds (Adj *p*-value < 0.05 and 2 < Ratio < 0.5). Compounds are ordered by their abundance, starting with the most abundant, based on the maximum area detected for each sample, as reported in the ‘Area max’ column; Figure S3. Individual and group TEWL percentage changes after week 1 (T0-T1w) following treatment with 0.5% *w*/*w* Hyssopus officinalis extract (HoEx) or placebo (*n* = 10). Statistical significance was assessed using a paired two-tailed Student’s *t*-test on the paired differences between HoEx- and placebo-treated sites (df = 9, *p* = 0.0071).

## Abbreviations

The following abbreviations are used in this manuscript:

CV	coefficient of variation
DA	differentially abundant
FLG	filaggrin
FOMT	flavonoid O-methyltransferase
GBA	β-glucocerebrosidase
HoEx	*Hyssopus officinalis* cell culture extract
INV	involucrin
LC-HESI-HRMS	liquid chromatography–heated electrospray–high resolution mass spectrometry
NMF	natural moisturizing factor
PCA	principal component analysis
PMF	polymethoxyflavone
RT	retention time
SC	stratum corneum
SPT	serine palmitoyltransferase
TEWL	transepidermal water loss
TIC	total ion current

## Appendix A

**Table A1:** Composition of the placebo and HoEx oil-in-water (O/W) emulsions used in the clinical study. The formulations were identical, except for the inclusion of 0.5% (*w*/*w*) HoEx in the active formulation. Ingredients are listed according to their INCI names, cosmetic function, and concentration (% *w*/*w*).

Ingredient (INCI Name)	Function	% *w*/*w*
AQUA (WATER)	solvent	up to 100
SODIUM GLUCONATE	chelating agent	0.20
HYSSOPUS OFFICINALIS EXTRACT	active ingredient	0.50
CARBOMER	gelling agent/viscosity control	0.70
GLYCERYL STEARATE (AND) PEG-100 STEARATE	emulsifying	4.00
CAPRYLIC/CAPRIC TRIGLYCERIDE	emollient	10.00
CETEARYL ALCOHOL	emulsion stabilising/viscosity controlling	0.50
CETYL RICINOLEATE	emollient	2.00
SODIUM HYDROXIDE	pH adjuster	0.10
PHENOXYETHANOL (AND) ETHYLHEXYLGLYCERIN	preservative system	0.90

**Table A2:** Main polyphenols accumulated in *Hyssopus officinalis* cell cultures grown in dark and light condition and in adult plants. Data are reported as Fold Internal Standard (IS, Formononetin) and represent AVG ± SD of three technical replicates. Asterisks indicate significant differences from the plant extract according to Student’s *t*-test (* *p* < 0.05, ** *p* < 0.01).

	Dark	Light	Plant
4-caffeoylquinic acid	0.0086 ± 0.0008 **	0.0174 ± 0.0004 **	4.4605 ± 0.2511
diosmin	0.0014 ± 0.0008 **	0.0188 ± 0.002 **	2.0652 ± 0.1687
5-caffeoylquinic acid	0.0048 ± 0.002 **	0.0047 ± 0.0003 **	0.5843 ± 0.0186
ferulic acid	0.0134 ± 0.0007 **	0.0087 ± 0.0012 **	0.5262 ± 0.0535
ursolic acid	0.0291 ± 0.0006 **	0.0128 ± 0.0009 **	0.6803 ± 0.031
syringic acid_1	0.0034 ± 0.0004 **	0.0011 ± 0.0002 **	0.0614 ± 0.0079
syringic acid_2	0.0089 ± 0.0007 *	0.0035 ± 0.0005 *	0.0777 ± 0.0165
caffeic acid	0.0038 ± 0.0004 **	0.0028 ± 0.0003 **	0.0599 ± 0.0075
diosmetin	0.0003 ± 0.00003 **	0.0002 ± 0.00004 **	0.0446 ± 0.0013
4-hydroxybenzoic acid	0.0502 ± 0.0051 *	0.0341 ± 0.0033 *	0.0152 ± 0.0045
hesperidin	0.0087 ± 0.001 **	0.0205 ± 0.0022 **	0.0005 ± 0.0003
3-caffeoylquinic acid	0.0067 ± 0.0004 *	0.0015 ± 0.0006 **	0.0141 ± 0.0013
methyl rosmarinic acid	0.0078 ± 0.0004 **	0.0049 ± 0.0001 *	0.0017 ± 0.0007
rosmarinic acid	2.8621 ± 0.1903 **	2.0496 ± 0.0962 **	0.5104 ± 0.023
rosmarinic acid glucoside isomer 2	0.4659 ± 0.0549 **	0.4404 ± 0.1977	0.0709 ± 0.009
rosmarinic acid glucoside isomer 1	0.191 ± 0.0139 **	0.1697 ± 0.0281 *	0.0269 ± 0.0012
