# Supplementary material for: Hyssopus officinalis L. (Lamiaceae) Cell Culture Extract Modulates Epidermal Lipid-Related and Differentiation Markers
Source: Cells. 2026 Jul 21;15(14):1300. doi: 10.3390/cells15141300 (PMC13406663; doi:10.3390/cells15141300)
Supplement: Supplementary file 1 [file cells-15-01300-s001.zip › cells-4224365-supplementary.pdf]

cells

MDPI

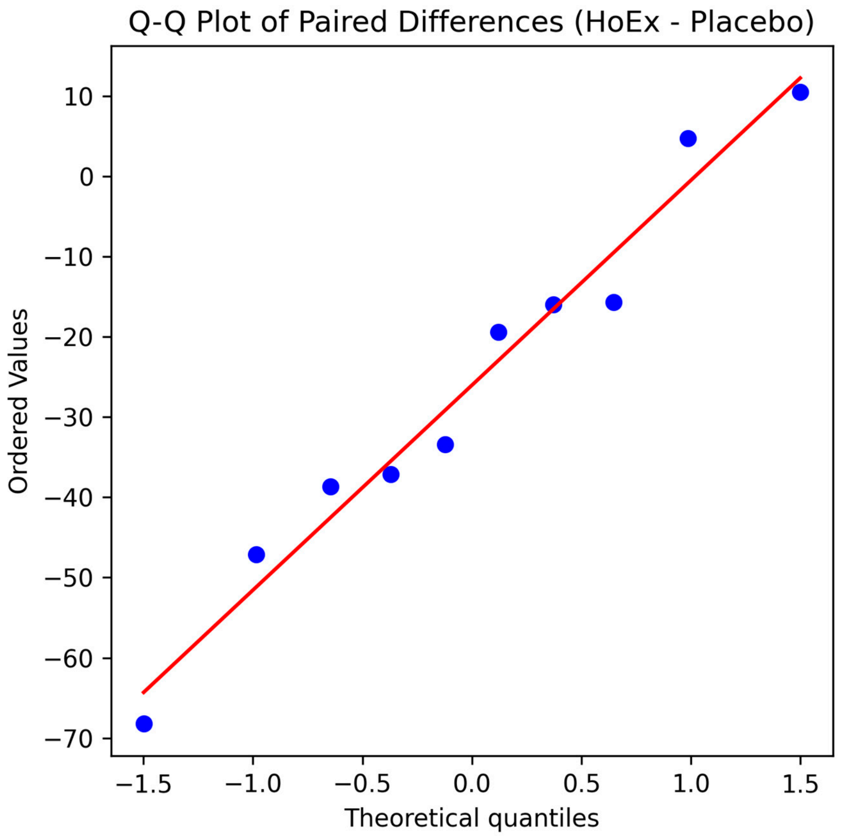

Figure S1. Q–Q plot of the paired differences in TEWL percentage change [Δ%(HoEx) − Δ%(Placebo)] obtained from the intra-individual comparison of skin sites treated with 0.5%w/w HoEx or placebo (n = 10). The distribution of paired differences closely followed the theoretical normal distribution, with no evident departures from linearity.

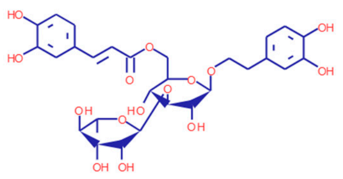

2-(3,4-Dihydroxyphenyl)ethyl 3-O-(6-deoxy-β-L-mannopyranosyl)-6-O-[(2E)-3-(3,4-dihydroxyphenyl)-2-propenoyl]-β-D-glucopyranoside

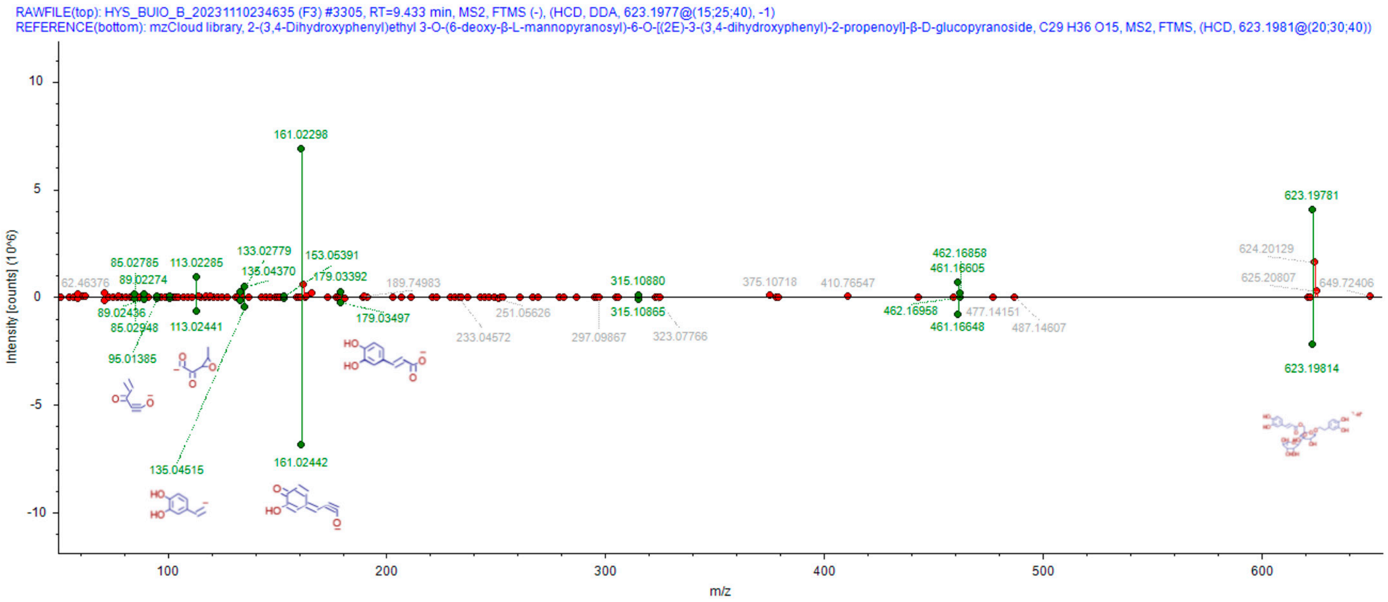

Figure S2. Compound detected at *m/z* 623.1971, identified as 2-(3,4-Dihydroxyphenyl)ethyl 3-O-(6-deoxy-β-L-mannopyranosyl)-6-O-[(2E)-3-(3,4-dihydroxyphenyl)-2-propenoyl]-β-D-glucopyranoside by match with mzCloud MS2 fragmentation pattern.

Table S1. LC-HESI-MS untargeted comparative analysis of semipolar compounds of *H. officinalis* plant and cell cultures, grown in dark (HoEx-D) and light conditions (HoEx-L), extracts cv. Levels of accumulation are reported as group area under the m/z peak of the preferred ion. Ratio HoEx-D/Plant and HoEx-L/Plant column cells are colored indicating significant differentially abundant compounds (Adj p-value<0.05 and 2<Ratio<0.5). Compounds are ordered by their abundance, starting with the most abundant, based on the maximum area detected for each sample, as reported in the 'Area max' column.

Name	Metabolic class	Formula	AnnotDelta Mass [ppm]	Calc. MW	m/z	RT [min]	Area (Max.)	Reference Ion	Group Area: HoEx-D	Group Area: HoEx-L	Group Area: plant	Group CV [%]: HoEx-D	Group CV [%]: HoEx-L	Group CV [%]: plant	Ratio: (HoEx-D / (plant)	Ratio: (HoEx-L) / (plant)	Adj. P-value: (HoEx-D) / (plant)	Adj. P-value: (HoEx-L) / (plant)
Rosmarinic acid	Hydroxycinnamic acids and derivatives	C18H16O8	-1.8	360.0839	361.0913	10.96	1.06E+10	[M+H-H2O]+1	1.04E+10	7.48E+09	1.68E+09	1.66	5.35	9.44	6.2032	4.4412	4.6E-07	1.3E-06
2-Hydroxy-3-{[(2E)-3-(4-hydroxyphenyl)-2-propenoyl]oxy}succinic acid	Hydroxycinnamic acids and derivatives	C13H12O8	-1.97	296.0526	295.0453	9.46	9.83E+09	[M-H]-1	1.81E+06	1.07E+06	8.71E+09	5.47	6.5	8.08	0.0002	0.0001	7.3E-13	7.5E-13
(7S,8S)-Syringoylglycerol-9-O-(6-O-cinnamoyl)-beta-D-glucopyranoside	Phenylpropanoids and glycosides	C26H32O12	-3.82	536.1887	554.2227	11.70	8.64E+09	[M+NH4]+1	9.90E+05	3.82E+06	7.29E+09	36.23	8.27	11.21	0.0001	0.0005	4.5E-08	2.2E-07
L-Norleucine	Amino acids and derivatives	C6H13NO2	1.61	131.0948	132.1021	1.81	5.43E+09	[M+H]+1	5.32E+09	5.34E+09	1.36E+09	3.14	2.59	6.72	3.8999	3.9154	3.6E-07	3.4E-07
3,6,19-Trihydroxyurs-12-en-28-oic acid	Triterpenoids	C30H48O5	-1.41	488.3495	489.3569	21.57	5.21E+09	[M+H]+1	4.76E+09	1.96E+09	8.87E+08	6.37	9.35	2.58	5.3602	2.2037	7.9E-07	2.3E-05

4-caffeoylquinic acid	Hydroxycinnamic acids and derivatives	C16H18O9	-1.19	354.0947	353.0873	7.03	4.84E+09	[M-H]-1	8.07E+06	1.82E+07	4.28E+09	5.66	2.34	7.88	0.0019	0.0043	7.3E-13	7.5E-13
hydroxytyrosol glucoside	Glycosides - Phenylethanoid -	C14H20O8	-1.36	316.1154	315.1081	4.75	4.80E+09	[M-H]-1	4.76E+09	4.18E+09	2.14E+09	2.29	3.71	5.75	2.2203	1.9498	2.8E-06	6.1E-06
Corosolic acid	Triterpenoids	C30H48O4	-1.07	472.3548	473.362	24.36	4.32E+09	[M+H]+1	4.18E+09	2.90E+09	1.38E+09	3.94	6.15	4.49	3.0364	2.1048	1.4E-06	9.0E-06
N-(1-Deoxy-1-fructosyl)leucine	Amino acids and derivatives	C12H23NO7	0.99	293.1477	294.155	1.90	3.91E+09	[M+H]+1	1.98E+09	3.89E+09	1.61E+07	1.85	1.22	42.37	122.7676	240.7388	3.7E-06	2.3E-06
1-O-caffeoyl-beta-xylose	Hydroxycinnamic acids and derivatives	C14H16O8	-0.97	312.0842	311.0769	8.07	3.59E+09	[M-H]-1	5.02E+05	9.50E+05	3.09E+09	20.7	35.65	11.29	0.0002	0.0003	2.1E-07	3.1E-07
Benzoyl-beta-D-glucoside	Glycosides - Phenolic -	C13H14O6	-1.69	285.0963	267.0859	10.01	3.08E+09	[M+H-H2O]+1	1.99E+06	6.68E+05	2.62E+09	29.71	21.75	10.38	0.0008	0.0003	2.1E-07	6.4E-08
Dehydrodicaffeic acid dilactone	Phenylpropanoic acid and derivatives	C18H14O8	-1.5	358.0683	357.061	10.90	3.01E+09	[M-H]-1	2.42E+09	1.99E+09	2.69E+08	13.25	30.21	9.38	9.0000	7.3949	5.3E-05	1.7E-04
Unknown		C24H33NO12	-0.69	527.1999	528.2072	10.02	2.97E+09	[M+H]+1	2.78E+05	9.29E+05	2.70E+09	31.53	48.98	6.17	0.0001	0.0003	4.3E-07	9.7E-07
4-Hydroxy-2-oxoglutaric acid	Others	C5H6O6	-2.83	162.016	323.0251	2.09	2.96E+09	[2M-H]-1	5.28E+05	6.33E+05	2.86E+09	4.2	6.47	4.07	0.0002	0.0002	7.3E-13	7.5E-13
Salvionic acid B	Phenylpropanoic acid and derivatives	C16H13N3O7	2.57	359.0763	717.1453	11.89	2.95E+09	[2M-H]-1	8.37E+08	2.23E+09	2.41E+09	9.09	2.59	11.93	0.3469	0.9253	1.4E-05	2.3E-01
Sucrose	Sugars	C12H22O11	-1.9	342.1156	387.1139	1.19	2.84E+09	[M+FA-H]-1	2.81E+09	1.91E+09	9.20E+08	1.19	13.98	24.96	3.0575	2.0734	8.8E-04	1.7E-02

Mono-trans-p-coumaroylmesotartaric acid	Hydroxycinnamic acids and derivatives	C14H14O8	-1.29	310.0685	309.0612	10.08	2.76E+09	[M-H]-1	1.37E+06	4.59E+06	2.43E+09	8.84	10.27	7.91	0.0006	0.0019	7.3E-13	7.6E-13
2-(3,4-Dihydroxyphenyl)ethyl 3-O-(6-deoxy-β-L-mannopyranosyl)-6-O-[(2E)-3-(3,4-dihydroxyphenyl)-2-propenoyl]-β-D-glucopyranoside	Glycosides - Phenylethanoid -	C29H36O15	-1.52	624.2045	623.1971	9.54	2.66E+09	[M-H]-1	2.04E+09	3.70E+08	1.38E+06	33.29	5.44	19.07	1478.6602	267.3841	2.7E-07	1.1E-06
Ipolamiide (phenylethanoids)	Glycosides - Phenylethanoid -	C17H26O11	-1.31	406.147	451.1451	2.48	2.65E+09	[M+FA-H]-1	3.97E+06	2.20E+06	2.59E+09	7.71	20.25	6.29	0.0015	0.0009	7.9E-10	1.4E-10
Diosmin	Flavonoids	C28H32O15	-0.56	608.1738	609.181	10.54	2.28E+09	[M+H]+1	1.04E+06	1.89E+07	1.99E+09	64.72	8.81	9.87	0.0005	0.0095	2.3E-06	2.5E-05
(7S,8S)-Syringoylglycerol-9-O-(6-O-cinnamoyl)-beta-D-glucopyranoside iso	Phenylpropanoids and glycosides	C26H35NO12	-1.04	536.1887	554.2226	11.08	2.22E+09	[M+NH4]+1	9.15E+05	7.82E+05	1.98E+09	5.59	2.85	6.7	0.0005	0.0004	7.3E-13	7.5E-13
Unknown		C22H32O10	-1.1	456.1991	457.2063	11.42	2.09E+09	[M+H]+1	1.63E+07	2.00E+07	1.89E+09	2.65	5.77	5.71	0.0086	0.0105	7.3E-13	7.5E-13
Unknown		C53H65N3O14P2	-0.25	1029.3939	1028.3866	8.83	2.06E+09	[M-H]-1	1.32E+06	8.70E+05	1.87E+09	1.25	0.85	5.92	0.0007	0.0005	7.3E-13	7.5E-13
12-Hydroxyjasmonic	Oxylipins and derivatives	C18H28O9	-1.11	388.1729	387.1656	7.67	1.98E+09	[M-H]-1	4.23E+05	6.03E+05	1.77E+09	3.52	18.16	7.53	0.0002	0.0003	7.4E-13	9.0E-13

acid 12-O-beta-D-glucoside																		
5-(3',4',5'-Trihydroxyphenyl)-γ-valerolactone	Pyrogallols and derivatives	C11H12O5	-3.64	224.0677	223.0604	7.96	1.94E+09	[M-H]-1	4.16E+07	1.27E+07	1.75E+09	5	4.36	13.24	0.0238	0.0072	8.6E-09	5.3E-11
5,4'-dihydroxy-3,3'-dimethoxy-6,7-methylenedioxyflavone	Flavonoids - polymethoxyflavones -	C18H14O8	-1.43	358.0684	359.0757	8.84	1.75E+09	[M+H]+1	1.70E+09	1.12E+09	1.23E+08	4.79	6.82	12.15	13.8199	9.0694	3.0E-07	8.5E-07
Thiamine	Vitamins	C12H16N4OS	-1.55	264.1041	265.1114	1.15	1.54E+09	[M+H]+1	1.38E+09	1.43E+09	9.75E+06	2.7	4.37	26.62	141.1810	146.8007	3.6E-07	3.4E-07
3-Methoxy-4-hydroxyphenylglycol glucuronide	Glucuronides	C15H20O10	-1.16	360.1052	359.098	4.08	1.53E+09	[M-H]-1	4.42E+07	2.48E+07	1.39E+09	14.12	8.88	5.94	0.0317	0.0178	1.3E-07	2.0E-08
Unknown		C4H6N6O4	-0.18	202.045	203.0523	1.19	1.43E+09	[M+H]+1	1.37E+09	1.25E+09	1.51E+08	2.8	4.92	26.68	9.0542	8.2225	1.0E-05	1.4E-05
Fertaric acid	Hydroxycinnamic acids and derivatives	C14H14O9	-1.46	326.0633	325.056	7.82	1.27E+09	[M-H]-1	3.24E+05	3.67E+05	1.16E+09	5.45	0.88	6.47	0.0003	0.0003	7.3E-13	7.5E-13
3,4-Methylenedioxycinnamaldehyde	Hydroxycinnamic acids and derivatives	C10H8O3	-1.91	176.047	177.0544	8.10	1.27E+09	[M+H]+1	1.33E+07	1.40E+07	1.13E+09	8.05	9.02	10.1	0.0118	0.0125	1.0E-09	1.2E-09
Adenosine	Nucleosides	C10H13N5O4	-2.13	267.0962	268.1035	1.26	1.24E+09	[M+H]+1	1.18E+09	1.07E+09	5.95E+07	5.4	2.74	35.72	19.7586	17.9067	1.1E-05	1.4E-05
4-(2-Carboxyethyl)-2-methoxyphenyl beta-D-glucopyranosiduronic acid	Others	C16H20O10	-1.55	372.1051	371.0978	8.67	1.18E+09	[M-H]-1	1.22E+08	1.88E+08	1.05E+09	1.38	2.95	8.54	0.1160	0.1795	1.3E-08	1.5E-07

3-[(1E,3E)-hepta-1,3-dienyl]pentanedioic acid	Carboxylic acids and derivatives	C12H18O4	-0.85	226.1203	227.1276	7.68	1.16E+09	[M+H]+1	8.28E+06	8.40E+06	1.03E+09	3.3	2.05	7.35	0.0080	0.0082	7.3E-13	7.5E-13
Unknown		C27H30N4O6P2	-1.55	568.1632	567.1559	2.44	1.16E+09	[M-H]-1	3.29E+06	1.20E+06	1.09E+09	4.21	35.98	4.39	0.0030	0.0011	5.9E-07	2.2E-07
3,3',7-Trimethylquercetin	Flavonoids - polymethylated flavonol -	C18H16O7	-1.51	344.0891	343.0818	12.17	1.13E+09	[M-H]-1	1.11E+09	8.62E+08	1.12E+08	9.35	13.47	10.55	9.8451	7.6757	2.4E-06	4.2E-06
1-O-Feruloyl-β-D-glucopyranose	Hydroxycinnamic acids and derivatives	C16H20O9	-1.76	356.1101	401.1083	6.97	1.13E+09	[M+FA-H]-1	1.06E+09	8.12E+08	8.33E+07	7.23	2.31	16.78	12.6795	9.7408	1.1E-06	1.7E-06
(E,E)-alpha-Farnesene	Sesquiterpenoids	C15H24	-1.08	204.1876	205.1949	23.71	1.11E+09	[M+H]+1	4.68E+05	2.65E+05	1.10E+09	23.98	38.03	1.32	0.0004	0.0002	1.1E-04	6.1E-05
Benzoyl glucuronide	Glucuronides	C13H14O8	-1.75	298.0683	297.061	6.36	1.08E+09	[M-H]-1	1.11E+06	6.30E+05	9.69E+08	18.46	21.77	7.03	0.0011	0.0007	3.4E-08	1.5E-08
Phenethyl beta-primeveroside	Glycosides - Phenolic -	C19H28O10	-0.71	416.168	434.2018	10.31	1.04E+09	[M+NH4]+1	1.98E+05	1.43E+05	9.59E+08	8.78	32.76	5.45	0.0002	0.0001	4.2E-09	2.8E-09
2-(D-glucosyloxy)benzoic acid	Others	C13H16O8	-1.58	300.084	299.0767	6.29	1.01E+09	[M-H]-1	2.78E+07	1.40E+07	9.13E+08	2.45	2.99	6.48	0.0305	0.0153	7.3E-13	7.5E-13
Unknown		C18H12O8	-1.27	356.0528	355.0455	12.24	9.50E+08	[M-H]-1	9.30E+08	5.02E+08	1.44E+08	1.99	4.82	8.37	6.4498	3.4847	1.8E-07	1.6E-06
Unknown		C43H42N4O9P2	-0.71	820.2421	819.2348	10.05	9.19E+08	[M-H]-1	2.48E+05	2.87E+05	8.95E+08	7.05	5.38	4.87	0.0003	0.0003	7.3E-13	7.5E-13
(10E,15Z)-9,12,13-Trihydroxy-10,15-octadecadienoic acid	Hydroxy Fatty acids and derivatves	C18H32O5	-1.37	328.2245	327.2172	14.51	8.61E+08	[M-H]-1	6.30E+08	8.34E+08	3.36E+08	2.55	2.66	4.73	1.8732	2.4812	3.3E-06	7.5E-07

Isoferulic acid	Hydroxycinnamic acids and derivatives	C10H10O4	0.25	194.058	195.0652	9.49	8.52E+08	[M+H]+1	4.24E+07	3.57E+07	5.76E+08	9.56	16.34	26.31	0.0737	0.0620	7.6E-06	5.1E-06
Benzyl alcohol beta-D-rutinoside	Glycosides - Phenolic -	C19H28O10	-0.78	416.1679	461.166	7.91	8.29E+08	[M+FA-H]-1	1.24E+06	1.05E+06	7.44E+08	35.44	16.14	11.45	0.0017	0.0014	8.3E-07	8.2E-07
(8E)-2-Amino-8-octadecene-1,3,4-triol	Others	C18H37NO3	-1.03	315.277	316.2843	16.28	8.16E+08	[M+H]+1	5.27E+08	6.43E+08	5.29E+07	2.33	14.76	2.91	9.9638	12.1571	6.2E-07	3.3E-07
[Similar to: (2R)-2-({(2E)-3-[3-{[(1R)-1-Carboxy-2-(3,4-dihydroxyphenyl)ethoxy]carbonyl}-2-(3,4-dihydroxyphenyl)-7-hydroxy-2,3-dihydro-1-benzofuran-4-yl]-2-propenoyl}oxy)-3-(3,4-dihydroxyphenyl) propanoic acid; ΔMass: 44.0253 Da]		C35H26N10O11	0.56	762.1787	761.1714	12.68	7.98E+08	[M-H]-1	1.26E+07	2.32E+07	7.29E+08	25.8	20.55	10.25	0.0173	0.0318	2.2E-06	3.8E-06
DL-Glutamine	Amino acids and derivatives	C5H10N2O3	-0.71	146.069	147.0763	1.22	7.96E+08	[M+H]+1	1.66E+08	2.02E+08	7.38E+08	9.1	8.33	14.77	0.2252	0.2734	1.6E-05	3.8E-05
2-(Benzoyloxy)-3-hydroxysuccinic acid	Carboxylic acids and derivatives	C11H10O7	-2.31	254.0421	253.0348	7.64	7.95E+08	[M-H]-1	1.69E+06	4.94E+05	7.88E+08	15.21	22.89	2.08	0.0021	0.0006	2.6E-08	2.1E-09

Unknown		C50H69N3O13P2	-0.78	981.4298	964.4265	8.69	7.91E+08	[M+H-H2O]+1	1.80E+05	1.66E+05	7.46E+08	3.24	8.56	4.7	0.0002	0.0002	7.3E-13	7.5E-13
LysoPC(18:3(9Z,12Z,15Z))	Glycerophospholipid	C26H48NO7P	-0.69	517.3165	518.3238	21.00	7.90E+08	[M+H]+1	1.07E+08	6.99E+08	1.10E+08	25.53	18.85	15.31	0.9773	6.3606	8.6E-01	1.6E-04
5,6,7-Trimethoxy-2H-chromen-2-one (or 5,6,7-Trimethoxycoumarin)	Coumarins and derivatives	C12H12O5	-2.98	236.0678	235.0604	11.19	7.84E+08	[M-H]-1	1.44E+06	1.38E+06	7.19E+08	8.79	5.35	6.33	0.0020	0.0019	7.3E-13	7.5E-13
(9Z,12Z,15Z)-2-Hydroxy-9,12,15-octadecatrienoic acid	Hydroxy Fatty acids and derivatves	C18H30O3	-1.3	294.2191	293.2119	22.22	7.78E+08	[M-H]-1	6.99E+08	3.07E+08	5.25E+07	6.64	4.41	3.46	13.3227	5.8546	2.1E-10	6.9E-08
Valine	Amino acids and derivatives	C5H11NO2	1.17	117.0791	118.0864	1.28	7.51E+08	[M+H]+1	6.95E+08	7.31E+08	2.95E+08	4.52	3.2	45.57	2.3579	2.4829	1.4E-02	1.2E-02
Unknown	Others	C15H26N2O3	-1.29	282.194	283.2013	8.06	7.49E+08	[M+H]+1	7.40E+08	5.78E+08	2.91E+05	0.73	1.92	7.29	2541.2266	1987.0797	7.3E-13	7.5E-13
Thiamine iso	Vitamins	C12H16N4OS	-1.5	264.1041	265.1114	0.95	7.35E+08	[M+H]+1	7.11E+08	6.37E+08	1.99E+06	2.65	2.68	2.21	356.4489	319.5366	7.3E-13	7.5E-13
Phenethyl beta-primeveroside iso	Glycosides - Phenylethanoid -	C19H28O10	-0.96	416.1679	434.2017	11.47	7.24E+08	[M+NH4]+1	2.06E+05	1.39E+05	6.63E+08	2.31	3.11	6.7	0.0003	0.0002	7.3E-13	7.5E-13
Unknown		C25H52NO9P	-1.46	541.3372	540.3299	23.38	7.15E+08	[M-H]-1	6.85E+08	5.56E+08	1.03E+08	15.19	13.26	5.97	6.6460	5.3983	7.2E-06	1.0E-05
Rosmarinic acid glucoside iso2	Hydroxycinnamic acids and derivatives	C23H25N8OP3	0.32	522.1366	521.1292	9.74	7.09E+08	[M-H]-1	4.25E+08	3.48E+08	7.46E+07	9.1	46.92	12.12	5.6949	4.6635	5.5E-04	5.4E-04

Palmitoyl lysophosphatidylethanolamine	Lysophospholipid	C21H44NO7P	-1.17	453.285	454.2924	23.24	6.95E+08	[M+H]+1	5.85E+08	3.49E+08	6.08E+07	14.21	3.01	1.13	9.6143	5.7378	5.4E-07	1.8E-06
Unknown		C9H16N4O2	-1.17	212.1271	254.1609	1.74	6.82E+08	[M+ACN+H]+1	6.80E+08	2.33E+08	9.42E+05	2.8	4.7	16.45	721.5438	247.0698	1.1E-12	7.4E-11
Unknown		C41H33N6O5P	1.27	720.2259	719.2187	12.62	6.78E+08	[M-H]-1	6.04E+08	5.72E+08	5.21E+05	10.01	7.42	9.26	1159.7657	1099.6972	7.3E-13	7.5E-13
L-Glutamic acid	Amino acids and derivatives	C5H9NO4	-0.99	147.053	148.0603	1.21	6.76E+08	[M+H]+1	6.73E+08	6.05E+08	2.03E+08	1.3	2.09	22.9	3.3192	2.9842	1.4E-04	2.2E-04
Unknown		C18H32O10	-0.83	408.1992	453.1973	9.23	6.60E+08	[M+FA-H]-1	2.47E+05	2.66E+05	5.82E+08	60.36	14.78	8.1	0.0004	0.0005	1.4E-06	1.3E-06
(2alpha,3beta,5xi)-2,3-Dihydroxyurs-12-en-28-oic acid	Triterpens	C30H48O4	-1.99	472.3543	517.3525	24.26	6.48E+08	[M+FA-H]-1	6.22E+08	6.36E+08	2.98E+08	0.19	3.89	11.69	2.0843	2.1323	6.9E-05	6.5E-05
6,8-Dihydroxy-7-methoxy-2H-chromen-2-one	Coumarins and derivatives	C10H8O5	-0.14	208.0371	209.0444	6.39	6.34E+08	[M+H]+1	2.18E+07	2.31E+06	5.56E+08	3	79.93	8.36	0.0393	0.0042	1.2E-02	4.8E-04
2,3,4-Trimethoxycinnamic Acid	Hydroxycinnamic acids and derivatives	C12H14O5	-3.41	238.0833	237.076	9.44	6.33E+08	[M-H]-1	1.40E+06	6.91E+05	5.32E+08	24.36	18.21	11.48	0.0026	0.0013	2.4E-07	1.3E-07
6,8-dihydroxy-7-methoxychromen-2-one	Coumarins and derivatives	C10H8O5	-2.13	208.0367	209.044	6.59	6.33E+08	[M+H]+1	2.18E+07	2.33E+06	5.54E+08	2.72	12.65	8.42	0.0394	0.0042	4.5E-08	3.4E-12
DL-Arginine	Amino acids and derivatives	C6H14N4O2	-1.3	174.1115	175.1187	1.22	6.22E+08	[M+H]+1	6.09E+08	4.36E+08	1.83E+08	6.23	2.52	13.44	3.3302	2.3871	1.4E-05	8.4E-05
Unknown		C18H29NO10	-1.32	419.1786	420.1859	7.74	6.18E+08	[M+H]+1	6.02E+08	2.34E+08	2.76E+06	1.54	7.53	25.42	218.0118	84.7838	1.3E-07	5.2E-07
D-(+)-Proline	Amino acids and derivatives	C5H9NO2	1.22	115.0635	116.0707	1.21	6.00E+08	[M+H]+1	1.49E+08	1.23E+08	5.52E+08	1.78	7.32	5.48	0.2699	0.2234	9.0E-07	4.8E-07

Methyl 3,5-diacetoxy-4-methoxybenzoate	Methoxybenzoic acids and derivatives	C13H14O7	-0.8	282.0737	623.1612	8.05	5.80E+08	[2M-H+HAc]-1	1.24E+07	1.26E+07	5.39E+08	2.99	21.95	7.08	0.0230	0.0233	6.0E-07	6.0E-07
1-Salicylate glucuronide	Glucuronides	C13H14O9	-1.24	314.0634	313.0561	6.70	5.69E+08	[M-H]-1	1.60E+06	7.63E+05	5.13E+08	32.47	1.6	6.76	0.0031	0.0015	2.4E-07	6.8E-08
Unknown		C27H28N4O9	-2.98	552.184	551.1765	9.18	5.66E+08	[M-H]-1	8.79E+05	2.48E+06	4.51E+08	59.39	6.86	14.32	0.0020	0.0055	2.8E-06	5.7E-06
Geranyl beta-primeveroside	Glycosides - Terpenoid -	C21H36O10	-0.9	448.2304	493.2286	12.87	5.58E+08	[M+FA-H]-1	9.95E+05	9.37E+05	5.30E+08	34.84	45.92	6.43	0.0019	0.0018	3.0E-06	2.8E-06
Unknown		C14H21NO8	-1.73	331.1261	332.1334	1.25	5.55E+08	[M+H]+1	5.43E+08	4.87E+08	2.63E+07	3.33	2.31	38.59	20.6855	18.5556	2.1E-05	2.6E-05
2-Deoxy-2-(pentanoylamino)-D-glucose	Sugars	C11H21NO6	0.45	263.137	264.1443	2.04	5.52E+08	[M+H]+1	2.69E+08	5.51E+08	3.43E+06	1.95	0.58	14.48	78.5862	160.8284	3.5E-10	9.4E-12
N-(1-Deoxy-1-fructosyl)valine	Amino acids and derivatives	C11H21NO7	-1.69	279.1313	280.1386	1.28	5.20E+08	[M+H]+1	2.96E+08	5.09E+08	2.28E+06	3.59	3.09	39.94	130.0348	223.1109	2.2E-06	1.6E-06
(1S,4S,5R,10S,13S,17S,19S,20R)-10-hydroxy-4,5,9,9,13,19,20-heptamethyl-24-oxahexacyclo[15.5.2.0Âa,Âaâ ¸.0â ´,Âaâ ·.0â µ,Âaâ ´.0â „,ÂaÂl]tetracos-15-en-23-one	Others	C30H46O3	-0.51	454.3445	455.3517	26.11	5.18E+08	[M+H]+1	4.76E+08	1.05E+08	7.66E+07	6.23	6.33	2.39	6.2175	1.3683	8.8E-08	6.2E-04
N-(1-Deoxy-1-fructosyl)valine iso	Amino acids and derivatives	C11H21NO7	-1.62	279.1314	280.1386	1.73	5.08E+08	[M+H]+1	2.43E+08	4.59E+08	7.03E+05	6.1	7.09	28.02	345.8154	653.6042	8.6E-08	2.9E-08

N-(3-Carboxypropanoyl)-5-hydroxynorvaline	Amino acids and derivatives	C9H15NO6	-2.37	233.0894	266.1228	1.22	4.93E+08	[M+H+MeOH]+1	4.71E+08	4.66E+08	2.86E+06	4.06	1.86	22.74	164.7967	163.2302	6.6E-08	6.9E-08
5-hydroxy-3-(4-methoxyphenyl)-7-[(3,4,5-trihydroxy-6-{[(3,4,5-trihydroxy-6-methyloxan-2-yl)oxy]methyl}oxan-2-yl)oxy]-4H-chromen-4-one	Flavonoids	C28H32O14	-0.68	592.1788	593.1859	12.38	4.90E+08	[M+H]+1	1.60E+07	1.52E+07	4.23E+08	8.69	8.06	9.49	0.0378	0.0359	3.7E-08	3.7E-08
Unknown		C26H33NO11	-1.27	535.2047	536.212	11.71	4.80E+08	[M+H]+1	2.14E+05	1.64E+05	4.27E+08	3.71	2.82	8.41	0.0005	0.0004	7.3E-13	7.5E-13
Isoleucine	Amino acids and derivatives	C6H13NO2	-0.29	131.0946	132.1019	1.31	4.80E+08	[M+H]+1	4.75E+08	4.49E+08	8.65E+07	0.88	2.52	21.71	5.4898	5.1922	1.4E-05	1.7E-05
Unknown		C53H61N7O8P2	-1.11	985.4046	986.4119	10.10	4.54E+08	[M+H]+1	1.39E+05	1.23E+05	3.98E+08	3.56	8.2	7.91	0.0003	0.0003	7.3E-13	7.5E-13
5,7-Dihydroxy-3-methoxyflavone	Flavonoids - polymethoxyflavones -	C16H12O5	-1.6	284.068	627.1499	14.65	4.38E+08	[2M-H+HAc]-1	3.45E+08	4.26E+08	1.44E+08	4.11	2.02	7.78	2.3977	2.9637	5.3E-06	2.0E-06
Unknown		C22H34O10	-0.34	458.215	459.2223	12.03	4.33E+08	[M+H]+1	3.58E+06	8.83E+06	4.31E+08	1.96	6.24	2.66	0.0083	0.0205	7.3E-13	7.5E-13
(15Z)-9,12,13-Trihydroxy-15-octadecenoic acid	Fatty acids and derivatves	C18H34O5	-1.34	330.2402	329.2329	15.39	4.19E+08	[M-H]-1	2.52E+08	4.12E+08	1.55E+08	1.2	1.93	3.31	1.6233	2.6574	1.8E-06	1.2E-08
Unknown		C27H44N4O5	-0.82	504.3308	505.3381	9.17	4.17E+08	[M+H]+1	4.12E+08	2.82E+08	3.91E+05	0.95	0.6	5.58	1055.1870	722.5334	7.3E-13	7.5E-13

(6,6-Dimethylbicyclo[3.1.1]hept-2-yl)methyl 6-O-[(2R,3R,4R)-3,4-dihydroxy-4-(hydroxymethyl)tetrahydro-2-furanyl]-β-D-glucopyranoside	Glycosides - Terpenoid -	C21H36O10	-0.51	448.2306	493.2286	12.53	4.15E+08	[M+FA-H]-1	3.22E+05	5.66E+05	3.66E+08	1.42	24.67	7.74	0.0009	0.0015	2.5E-09	9.0E-09
N-{4-[(2,3-Dihydroxybenzoyl)amino]butyl}-N-{3-[(2,3-dihydroxybenzoyl)amino]propyl}-N'-hydroxy-N'-methylsuccinamide	Others	C26H34N4O9	-3.13	546.2309	545.2235	13.13	4.07E+08	[M-H]-1	2.42E+05	4.18E+05	3.60E+08	2.33	3.71	8.69	0.0007	0.0012	7.3E-13	7.5E-13
D-ribosylnicotinate	Others	C11H13NO6	-1.71	255.0739	256.0811	1.23	4.03E+08	[M+H]+1	3.98E+08	2.63E+08	1.82E+07	3.93	4.14	19.7	21.9308	14.4629	8.2E-07	1.5E-06
Unknown		C6H15O10P	-2.26	278.0397	277.0323	1.21	4.01E+08	[M-H]-1	2.27E+08	3.95E+08	2.75E+06	3.33	1.46	10.41	82.3386	143.2516	7.6E-13	7.5E-13
Salicylic acid glucoside	Glycosides - Phenolic -	C13H16O8	-1.61	300.084	299.0768	5.41	3.52E+08	[M-H]-1	3.50E+08	1.81E+08	1.20E+07	5.12	2.71	55.54	29.1238	15.0608	5.5E-05	1.9E-04
NP-007494	Others	C32H42O16	-0.18	682.2472	727.2452	8.11	3.42E+08	[M+FA-H]-1	2.06E+05	4.18E+05	3.05E+08	0.95	25.23	9.04	0.0007	0.0014	6.4E-09	2.1E-08
L-Tyrosine	Amino acids and derivatives	C9H11NO3	-0.67	181.0738	182.081	1.29	3.12E+08	[M+H]+1	3.01E+08	3.01E+08	3.06E+07	3.19	2.31	49.16	9.8326	9.8460	2.6E-04	2.6E-04

α-Eleostearic acid	Fatty acids and derivatves	C18H30O2	-1.42	278.2242	279.2315	23.78	3.07E+08	[M+H]+1	2.81E+08	1.55E+08	4.26E+07	7.14	5.75	13.07	6.5971	3.6361	1.8E-06	8.4E-06
cis-cinnamic acid	Phenylpropanoids and glycosides	C9H8O2	-0.21	148.0524	131.0491	11.08	2.98E+08	[M+H-H2O]+1	1.11E+06	6.68E+05	2.57E+08	10.98	2.85	9.6	0.0043	0.0026	1.7E-12	7.5E-13
2-Phenylethyl 6-O-(6-deoxy-alpha-L-mannopyranosyl)-beta-D-glucopyranoside	Phenylethanoids	C20H30O10	-1.17	430.1834	475.1815	8.98	2.97E+08	[M+FA-H]-1	5.41E+05	3.81E+05	2.69E+08	13.06	10.77	6.43	0.0020	0.0014	1.3E-11	5.0E-12
1-Salicylate glucuronide	Glucuronides	C13H14O9	-1.27	314.0634	313.0562	5.42	2.85E+08	[M-H]-1	7.82E+05	7.23E+05	2.65E+08	3.54	3.73	4.7	0.0030	0.0027	7.3E-13	7.5E-13
(4S)-4-hydroxy-3,5,5-trimethyl-4-[(1E)-3-{[(2R,3R,4S,5S,6R)-3,4,5-trihydroxy-6-(hydroxymethyl)oxan-2-yl]oxy}but-1-en-1-yl]cyclohex-2-en-1-one	Others	C19H30O8	-0.24	386.194	387.2013	7.91	2.77E+08	[M+H]+1	3.57E+06	3.93E+06	2.70E+08	1.72	3.29	7.52	0.0132	0.0145	7.3E-13	7.5E-13
2-(3,4-Dihydroxyphenyl)ethyl 3-O-(6-deoxy-β-L-mannopyranosyl)-6-O-[(2E)-3-(3,4-dihydroxyphenyl)-	Phenylethanoids	C29H36O15	-1.2	624.2047	623.1973	10.32	2.61E+08	[M-H]-1	2.12E+08	4.40E+07	8.90E+06	14.71	14.95	10.37	23.8382	4.9442	1.0E-06	2.3E-05

2-propenoyl]-β-D-glucopyranoside																		
Benzoyl glucuronide iso	Glucuronides	C13H14O8	-1.69	298.0684	297.061	7.61	2.55E+08	[M-H]-1	2.33E+05	4.29E+05	2.30E+08	16	30.34	7.4	0.0010	0.0019	2.4E-07	3.8E-07
Unknown		C21H36N2O8	-1.1	444.2467	467.2359	7.45	2.55E+08	[M+Na]+1	2.51E+08	2.35E+08	1.56E+05	8.48	8.11	5.11	1607.0968	1501.9546	7.3E-13	7.5E-13
N-(1-Deoxy-1-fructosyl)methionine	Amino acids and derivatives	C11H21NO7S	-1.83	311.1033	312.1106	1.74	2.53E+08	[M+H]+1	1.26E+08	2.50E+08	6.86E+05	2.73	4.13	39.92	182.9013	364.2267	1.8E-06	1.2E-06
1,2-Dibutyl citrate	Carboxylic acids and derivatives	C14H24O7	-0.49	304.1521	322.1859	7.01	2.41E+08	[M+NH4]+1	4.20E+05	7.11E+05	2.20E+08	57.28	5.3	7.15	0.0019	0.0032	2.2E-06	2.7E-06
12-Oxo phytodienoic acid	Oxylipins and derivatives	C18H28O3	-1.71	292.2033	293.2106	22.96	2.30E+08	[M+H]+1	2.25E+08	9.68E+07	4.17E+06	2.47	1.58	23.59	53.9392	23.2179	5.4E-07	1.5E-06
Caffeic acid 3-glucoside	Glycosides - Hydroxycinnamic -	C15H18O9	-1.41	342.0946	341.0873	5.85	2.22E+08	[M-H]-1	1.60E+08	2.11E+08	3.61E+07	4.4	5.3	9.37	4.4251	5.8547	1.5E-06	7.3E-07
5-hydroxy-3-(4-hydroxyphenyl)-7-{[(2S,3R,4S,5S,6R)-3,4,5-trihydroxy-6-({[(2R,3R,4R,5R,6S)-3,4,5-trihydroxy-6-methyloxan-2-yl]oxy}methyl)oxan-2-yl]oxy}-4H-chromen-4-one	Others	C27H30O14	-1.03	578.163	579.1704	10.17	2.19E+08	[M+H]+1	1.78E+06	4.78E+06	1.89E+08	12.78	9.85	9.02	0.0094	0.0253	6.2E-09	1.2E-07
(2S,3R,4S,5S,6R)-3,4,5-trihydroxy-6-	Others	C15H18O7	-2.07	310.1046	311.1119	11.70	2.18E+08	[M+H]+1	2.79E+05	2.84E+05	2.15E+08	28.8	28.7	1.42	0.0013	0.0013	6.3E-07	5.7E-07

(hydroxymethyl)oxan-2-yl (2E)-3-phenylprop-2-enoate																		
NP-003191		C27H30O15	-0.9	594.1579	593.1506	9.34	2.16E+08	[M-H]-1	3.74E+06	1.22E+07	2.10E+08	4.83	3.53	6.07	0.0178	0.0584	7.3E-13	5.3E-11
Unknown		C18H18O11	-1.8	410.0842	411.0915	10.50	2.16E+08	[M+H]+1	2.05E+08	1.81E+07	1.75E+05	3.56	19.27	40.71	1172.0355	103.6414	1.0E-06	4.6E-06
Guanine	Nucleotides	C5H5N5O	-1.42	151.0492	152.0565	1.23	2.15E+08	[M+H]+1	6.01E+07	1.99E+08	3.84E+06	2.72	7.09	44.57	15.6310	51.6731	9.0E-05	1.4E-05
2-(beta-D-Glucopyranosyloxy)-5-hydroxybenzoic acid	Glycosides - Phenolic -	C13H16O9	-1.08	316.0791	315.0718	7.89	2.09E+08	[M-H]-1	1.58E+05	2.07E+05	1.83E+08	4.7	2.68	10.62	0.0009	0.0011	7.3E-13	7.5E-13
Unknown		C13H14O5	-1.17	250.0838	251.0911	10.01	2.00E+08	[M+H]+1	6.23E+05	6.22E+05	1.68E+08	28.89	62.35	10.76	0.0037	0.0037	3.1E-05	2.3E-05
Rosmarinic acid glucoside iso1	Hydroxycinnamic acids and derivatives	C20H22N6O11	3.79	522.1366	521.1293	9.24	1.98E+08	[M-H]-1	1.73E+08	1.84E+08	2.65E+07	4.63	15.13	5.4	6.5278	6.9458	2.5E-06	2.6E-06
Vanillin	Others	C8H8O3	-2.12	152.047	153.0543	8.84	1.95E+08	[M+H]+1	3.18E+07	1.33E+07	1.92E+08	24.57	24.57	8.36	0.1657	0.0692	1.6E-04	1.9E-05
N-(1-Deoxy-1-fructosyl)tyrosine	Amino acids and derivatives	C15H21NO8	-1.52	343.1262	344.1335	1.74	1.90E+08	[M+H]+1	1.03E+08	1.82E+08	4.43E+05	3.57	5.33	10.16	232.9286	410.2394	7.3E-13	7.5E-13
(3S)-3-{[(3S)-3-Aminobutanoyl]amino}-5-methylhexanoic acid	Amino acids and derivatives	C11H22N2O3	-1.97	230.1626	231.1699	4.70	1.89E+08	[M+H]+1	1.85E+08	1.47E+08	2.32E+06	1.39	1.19	4.6	79.7691	63.2670	7.3E-13	7.5E-13

N-(1-Deoxy-1-fructosyl)tryptophan	Amino acids and derivatives	C17H22N2O7	-1.7	366.1421	367.1493	4.89	1.87E+08	[M+H]+1	9.91E+07	1.81E+08	3.23E+06	1.68	2.16	45.09	30.6572	55.8982	1.8E-05	7.1E-06
Desoxyrhaponticin	Others	C21H24O8	-1.49	404.1465	403.1392	12.01	1.86E+08	[M-H]-1	1.72E+08	3.61E+07	8.52E+05	5.05	2.43	36.61	201.6299	42.3530	1.2E-06	4.6E-06
Unknown		C10H16N2O4	-1.75	228.1106	261.1441	1.73	1.85E+08	[M+H+MeOH]+1	1.81E+08	1.36E+08	3.82E+05	2.77	7.55	2.13	473.3767	355.4280	7.3E-13	7.5E-13
unknown		C16H25N3O2	-2.52	291.1939	292.2012	8.67	1.83E+08	[M+H]+1	1.78E+05	1.65E+05	1.48E+08	3.24	8.56	13.2	0.0012	0.0011	7.3E-13	7.5E-13
4',6-Dihydroxy-5,7-dimethoxyflavone	Flavonoids - polymethoxyflavones -	C17H14O6	-1.59	314.0785	315.0858	13.96	1.77E+08	[M+H]+1	1.74E+08	1.49E+08	3.40E+07	5.55	7.54	2.98	5.1163	4.3730	4.9E-07	8.9E-07
N-(2-Phenylethyl)-beta-D-glucopyranuronosylamine	Others	C14H19NO6	-1.62	297.1208	298.128	3.41	1.69E+08	[M+H]+1	7.51E+07	1.68E+08	1.87E+06	0.8	1.57	11.03	40.1810	89.9277	2.8E-11	7.5E-13
Unknown		C24H42O6	-1.08	426.2977	444.3315	24.62	1.68E+08	[M+NH4]+1	4.45E+07	9.95E+07	1.66E+08	4.41	2.06	1.24	0.2679	0.5986	4.6E-09	3.2E-06
12-Oxo phytodienoic acid iso	Oxylipins and derivatives	C18H28O3	-1.43	292.2034	293.2107	23.29	1.63E+08	[M+H]+1	1.54E+08	1.30E+08	6.27E+07	5.06	6.5	2.71	2.4526	2.0786	3.0E-06	9.4E-06
(2R)-1-{[(2-Aminoethoxy)(hydroxy)phosphoryl]oxy}-3-hydroxy-2-propanyl (9E,12E)-9,12-octadecadienoate	Others	C23H44NO7P	-1.06	477.285	478.2925	21.84	1.59E+08	[M+H]+1	3.61E+07	3.26E+07	1.47E+08	1.92	4.21	10.96	0.2460	0.2221	2.1E-06	1.8E-06

Salidroside ISO	Phenylpropanoids and glycosides	C14H20O7	-1.56	300.1204	345.1187	5.83	1.58E+08	[M+FA-H]-1	1.37E+08	3.58E+07	1.46E+08	1.51	1.94	5.06	0.9362	0.2453	4.9E-02	6.3E-09
L-gamma-Glutamyl-L-leucine	Amino acids and derivatives	C11H20N2O5	-1.59	260.1368	261.1441	3.05	1.51E+08	[M+H]+1	1.50E+08	1.04E+08	1.35E+06	0.9	1.18	7.92	110.9668	76.8145	7.3E-13	7.5E-13
1-alpha-linolenoyl-sn-glycerol	Glicerides	C21H36O4	-1.21	352.2609	353.2682	20.20	1.48E+08	[M+H]+1	1.15E+07	6.83E+06	1.36E+08	15.28	1.42	5.82	0.0844	0.0502	7.3E-07	3.0E-07
9S,13R-12-Oxophytodienoic acid	Others	C18H28O3	-1.24	292.2035	293.2108	14.51	1.23E+08	[M+H]+1	9.10E+07	1.21E+08	5.66E+07	0.94	0.81	6.57	1.6072	2.1416	2.8E-05	2.4E-06
3,6-Dihydroxy-7-methoxy-2H-chromen-2-one (3-Hydroxyisoscopoletin)	Coumarins and derivatives	C10H8O5	-3.01	208.0366	253.0348	2.91	1.22E+08	[M+FA-H]-1	3.79E+05	4.49E+05	1.21E+08	1.61	4.96	3.3	0.0031	0.0037	7.3E-13	7.5E-13
9(S)-HpOTrE	Fatty acids and derivatves	C18H30O4	-1.36	310.214	309.2067	16.92	1.18E+08	[M-H]-1	1.17E+08	3.31E+07	2.96E+06	2.52	4.77	8.87	39.5642	11.1899	7.8E-12	2.4E-08
Diisodityrosine	Amino acids and derivatives	C36H38N4O12	-0.31	718.2484	741.2382	10.01	1.18E+08	[M+Na]+1	1.22E+05	1.02E+05	1.01E+08	4.46	8.2	10.14	0.0012	0.0010	7.3E-13	7.5E-13
2-(3,4-Dimethoxyphenyl)-5-hydroxy-3,7,8-trimethoxy-4H-chromen-4-one	Flavonoids - polymethoxyflavones -	C20H20O8	-0.76	388.1155	389.1228	10.30	1.01E+08	[M+H]+1	6.22E+07	6.38E+07	1.64E+06	2.75	30.5	36.67	37.9544	38.9702	1.6E-05	1.3E-05
7-Hydroxy-6-[(1E)-3-oxo-1-buten-1-yl]-2H-chromen-2-one	Coumarins and derivatives	C13H10O4	-0.94	230.0577	231.065	10.01	9.64E+07	[M+H]+1	1.67E+05	1.39E+05	7.93E+07	1.97	8.2	14.35	0.0021	0.0018	1.2E-12	9.0E-13

5,7-Dihydroxy-2-(4-hydroxyphenyl)-6,8-bis[3,4,5-trihydroxy-6-(hydroxymethyl)tetrahydro-2H-pyran-2-yl]-4H-chromen-4-one	Flavonoids	C27H30O15	-0.57	594.1581	595.1656	7.70	9.24E+07	[M+H]+1	1.76E+05	1.38E+05	8.03E+07	2.83	0.72	8.29	0.0022	0.0017	7.3E-13	7.5E-13
4-Coumaric acid	Phenolic acid	C9H8O3	-1.13	164.0472	147.0439	11.75	9.19E+07	[M+H-H2O]+1	6.99E+06	8.28E+06	8.71E+07	4.86	11.54	8.96	0.0803	0.0951	5.5E-07	7.4E-07
Tetraacetylethylene diamine	Others	C10H16N2O4	-2.07	228.1105	246.1444	2.53	8.40E+07	[M+NH4]+1	8.34E+07	7.67E+07	7.83E+05	1.52	13.44	6.51	106.5717	97.9972	3.6E-10	7.2E-10
rac-Anhalonidine	Others	C12H17NO3	-1.21	223.1206	265.1544	6.00	8.37E+07	[M+ACN+H]+1	8.35E+07	6.62E+07	1.22E+06	3.07	2.92	13.59	68.6518	54.4985	4.3E-10	1.2E-09
Valerophenone	Others	C11H14O	-1.57	162.1042	163.1115	9.00	8.14E+07	[M+H]+1	7.91E+07	4.40E+06	6.53E+06	4.48	10.26	11.5	12.1156	0.6742	6.4E-07	1.1E-02
4',7-Dihydroxyflavanone	Flavonoids	C15H12O4	-2.01	256.073	257.0803	11.69	7.98E+07	[M+H]+1	2.14E+05	1.64E+05	6.65E+07	3.71	2.82	12.42	0.0032	0.0025	7.3E-13	7.5E-13
Octahydro-1H-isoindole-1-carboxylic acid	Carboxylic acids and derivatives	C9H15NO2	-1.25	169.1101	170.1173	7.39	7.81E+07	[M+H]+1	4.89E+07	7.30E+07	1.03E+06	6.05	4.01	63.71	47.3854	70.8022	1.4E-04	8.3E-05
Verimol B	Methoxybenzoic acids and derivatives	C18H20O5	-0.33	316.131	317.1382	8.25	7.63E+07	[M+H]+1	8.64E+04	8.44E+04	6.84E+07	2.87	4.7	6.85	0.0013	0.0012	7.3E-13	7.5E-13
3-Amino-2,3,6-trideoxy-6-[[(1S)-1-[(3S)-3,4-dihydro-	Others	C20H28N2O8	-1.47	424.1839	425.1912	6.97	7.62E+07	[M+H]+1	2.96E+07	7.26E+07	4.93E+05	8.02	3.73	3.68	60.1403	147.2741	7.3E-13	7.5E-13

8-hydroxy-1-oxo-1H-2-benzopyran-3-yl]-3-methylbutyl]amino]-6-oxo-D-ribo-hexonic acid																		
Salidroside	Phenylpropanoids and glycosides	C14H20O7	-1.42	300.1205	299.1132	16.86	7.39E+07	[M-H]-1	2.66E+05	4.08E+05	7.31E+07	5.34	4.02	4.94	0.0036	0.0056	7.3E-13	7.5E-13
4,5,7,12-tetrahydroxy-11-methyl-6-methylidene-16-oxo-15-oxapentacyclo[9.3.2.15,8.01,10.02,8]heptadecane-9-carboxylic acid	Others	C19H24O8	-0.77	380.1468	381.1541	10.31	7.32E+07	[M+H]+1	1.95E+05	1.34E+05	6.73E+07	3.34	3.68	5.23	0.0029	0.0020	7.3E-13	7.5E-13
Caffeic acid	Hydroxycinnamic acids and derivatives	C9H8O4	-1.1	180.0421	181.0494	7.03	6.98E+07	[M+H]+1	3.34E+06	2.92E+06	5.54E+07	8.56	8.16	14.82	0.0603	0.0528	6.6E-07	4.9E-07
Tetrahydroharman-3-carboxylic acid	Alkaloids	C13H14N2O2	-1.27	230.1052	231.1126	6.83	6.90E+07	[M+H]+1	2.93E+07	6.70E+07	1.62E+05	2.17	2.34	5.68	180.6493	412.8498	7.3E-13	7.5E-13
5-Hydroxy-3,7-dimethoxy-3',4'-methylenedioxyflavone	Flavonoids - polymethoxyflavones -	C18H14O7	-1.46	342.0735	341.0662	12.04	6.34E+07	[M-H]-1	6.32E+07	3.52E+07	1.57E+06	3.08	3.85	30.44	40.1874	22.3822	2.2E-06	4.3E-06

2'-Hydroxy 3,6,7,4'-tetramethylquercetagetin	Flavonoids - polymethylated flavonol -	C19H18O9	-1.19	390.0946	391.1018	12.98	5.56E+07	[M+H]+1	1.20E+05	8.49E+04	5.17E+07	4.28	3.86	5.06	0.0023	0.0016	7.3E-13	7.5E-13
(2Z,4E)-2,4-Pentadecadienal	Others	C15H26O	-2.12	222.1979	205.1947	16.86	5.43E+07	[M+H-H2O]+1	7.95E+05	1.53E+05	5.41E+07	56.86	64.73	2.12	0.0147	0.0028	2.0E-04	6.7E-05
5'-Deoxy-5'-(methylsulfinyl)adenosine	Others	C11H15N5O4S	-2.52	313.0837	346.1172	1.73	5.42E+07	[M+H+MeOH]+1	4.86E+07	4.72E+07	3.48E+05	7.64	14.29	5.3	139.4676	135.6141	1.0E-09	1.6E-09
2-[4-(Methylsulfanyl)butyl]-3-oxosuccinate	Others	C9H12O5S	-2.79	232.0399	231.0325	5.04	5.32E+07	[M-H]-1	6.91E+06	5.15E+07	4.85E+05	4.46	2.06	4.07	14.2571	106.2673	7.3E-13	7.5E-13
Methyl 3-amino-2,3,6-trideoxyhexopyranoside	Others	C7H15NO3	-1.73	161.1049	203.1388	1.75	5.18E+07	[M+ACN+H]+1	4.99E+07	3.38E+07	4.79E+05	3.2	2.71	5.3	104.1246	70.4825	7.3E-13	7.5E-13
4'-O-beta-D-glucosyl-cis-p-coumaric acid	Hydroxycinnamic acids and derivatives	C15H18O8	-0.84	326.0999	327.1076	8.40	5.13E+07	[M+H]+1	2.96E+05	2.30E+05	5.06E+07	5.62	23.66	2.04	0.0058	0.0045	4.2E-08	3.0E-08
8-Epiiridodial glucoside	Glycosides - Terpenoid -	C16H26O7	-2.24	330.1671	331.1746	8.90	4.87E+07	[M+H]+1	3.28E+05	5.25E+05	4.76E+07	4.25	42.33	1.62	0.0069	0.0110	2.3E-06	3.4E-06
Diosmetin	Flavonoids	C16H12O6	-1.3	300.063	301.0704	14.70	4.61E+07	[M+H]+1	2.72E+05	2.23E+05	4.41E+07	12.52	18.54	3.66	0.0062	0.0051	2.3E-08	1.7E-08
Luteolin 6-C-glucoside 8-C-arabinoside	Flavonoids	C27H30O16	-0.3	610.1532	609.1459	12.04	4.43E+07	[M-H]-1	7.30E+06	1.22E+07	4.14E+07	3.93	3.51	5.13	0.1765	0.2944	1.6E-08	3.8E-07
Glutamylphenylalanine	Amino acids and derivatives	C14H18N2O5	-2.29	294.1209	295.1281	2.47	4.01E+07	[M+H]+1	3.86E+07	2.23E+07	7.39E+05	8.31	3.48	6.51	52.2240	30.1752	9.6E-12	3.2E-10
5,7-Dihydroxy-2-(4-hydroxy-2-	Flavonoids	C21H22O6	-0.07	370.1416	371.1489	8.35	3.89E+07	[M+H]+1	9.75E+04	2.30E+06	3.56E+07	21.98	14.42	7.12	0.0027	0.0648	4.2E-08	3.0E-06

methoxyphenyl)-8-(3-methyl-2-buten-1-yl)-2,3-dihydro-4H-chromen-4-one																		
Unknown		C24H40O6	-1.71	424.2818	425.2891	25.35	3.76E+07	[M+H]+1	1.65E+07	3.40E+07	5.32E+05	12.94	8.28	3.85	31.0332	63.8716	7.4E-08	5.6E-09
Syringaresinol-di-O-glucoside	Lignans	C34H46O18	-0.9	742.2677	760.3014	8.58	3.74E+07	[M+NH4]+1	2.62E+06	3.46E+07	1.48E+07	3.57	8.49	11.65	0.1772	2.3442	2.0E-06	7.5E-05
Geranyl glucoside	Glycosides - Terpenoid -	C16H28O6	-1.02	316.1883	317.1956	12.90	3.47E+07	[M+H]+1	1.59E+05	1.27E+05	2.97E+07	8.32	9.38	9.41	0.0053	0.0043	2.5E-11	5.6E-12
Geranyl glucoside iso	Glycosides - Terpenoid -	C16H28O6	-0.13	316.1886	317.1958	12.53	3.43E+07	[M+H]+1	1.40E+05	1.39E+05	3.10E+07	5.91	35	6.1	0.0045	0.0045	4.9E-07	6.3E-07
4-Caffeoylshikimic acid	Hydroxycinnamic acids and derivatives	C16H16O8	3.13	336.0856	714.2052	10.50	3.07E+07	[2M+ACN+H]+1	2.80E+07	9.86E+06	6.07E+04	5.58	19.01	5.89	461.7572	162.4387	1.6E-10	4.2E-09
3,8-Dimethoxy-5,7-dihydroxy-3',4'-methylenedioxyflavone	Flavonoids - polymethoxyflavones -	C18H14O8	-1.39	358.0684	717.1439	11.87	2.76E+07	[2M+H]+1	8.54E+06	2.40E+07	2.64E+07	13.1	8.17	2.86	0.3239	0.9107	1.8E-05	7.0E-01
Pinocamphone	Monoterpenoids	C10H16O	-0.81	152.12	153.1273	7.40	2.69E+07	[M+H]+1	3.01E+06	2.71E+06	2.63E+07	7.52	2.41	2.4	0.1144	0.1028	4.0E-09	2.0E-09
(3R)-7-Hydroxy-3-(2-hydroxy-4,5-dimethoxyphenyl)-2,3-dihydro-4H-chromen-4-one	Others	C17H16O6	-0.95	316.0944	315.0871	13.11	2.49E+07	[M-H]-1	5.15E+05	2.46E+07	4.55E+05	20.42	2.97	12.95	1.1301	53.9948	7.0E-01	5.2E-07
Hesperidin	Flavonoids	C28H34O15	-0.57	610.1894	609.1822	10.84	2.34E+07	[M-H]-1	8.11E+06	2.11E+07	3.70E+05	8.09	9.58	61.45	21.9407	56.9797	1.8E-04	3.6E-05

Tetrahydroharman-3-carboxylic acid	Alkaloids	C13H14N2O2	-1.39	230.1052	231.1125	7.27	1.92E+07	[M+H]+1	6.76E+06	1.81E+07	2.45E+05	2.7	3.6	5	27.5301	73.7055	7.3E-13	7.5E-13
5-[4,5-dihydroxy-6-(hydroxymethyl)-3-(3,4,5-trihydroxyoxan-2-yl)oxyoxan-2-yl]oxy-7,8-dimethoxy-3-(4-methoxyphenyl)chromen-4-one	Flavonoids	C29H34O15	-0.63	622.1894	623.1967	11.59	1.90E+07	[M+H]+1	7.48E+06	8.20E+05	1.66E+07	1.88	17.48	9.55	0.4500	0.0493	3.6E-04	6.8E-07
2-(2-amino-3-methylbutanamido)-3-phenylpropanoic acid	Others	C14H20N2O3	-1.83	264.1469	263.1397	6.00	1.86E+07	[M-H]-1	1.81E+07	1.49E+07	3.65E+05	2.3	2.34	4.78	49.4996	40.6888	7.3E-13	7.5E-13
Ferulic acid 4-glucuronide	Glucuronides	C16H18O10	-1.79	370.0893	393.0785	6.60	1.82E+07	[M+Na]+1	1.75E+07	1.81E+06	2.33E+05	2.89	4.19	6.81	75.0522	7.7873	7.3E-13	1.2E-08
Methyl rosmarinic acid	Hydroxycinnamic acids and derivatives	C19H18O8	-1.79	374.0995	375.1068	13.43	7.16E+06	[M+H]+1	7.10E+06	5.13E+06	1.47E+06	0.96	1.04	43.17	4.8311	3.4902	9.1E-04	3.4E-03

Figure S3. Individual and group TEWL percentage changes after week 1 (T0-T1w) following treatment with 0.5% w/w *Hyssopus officinalis* extract (HoEx) or placebo (n = 10). Statistical significance was assessed using a paired two-tailed Student's t-test on the paired differences between HoEx- and placebo-treated sites (df = 9, p = 0.0071).

Tewl T0 - T1w (paired t-test)			
	Hyssopus (HyEx) 0,5%	Placebo	Δ% (HoEx - Δ% (Placebo)
	-6,3	30,8	-37,0
	-2,4	-7,1	4,7
	-13,4	2,6	-16,0
	-8,9	10,5	-19,5
	-2,0	36,7	-38,7
	-48,4	-15,0	-33,4
	-14,3	53,9	-68,2
	2,9	50,0	-47,1
	18,1	7,6	10,5
	-9,6	6,1	-15,7
Media gruppo	-8,4	17,6	-26,0
Std. Dev			23,8
Numero soggetti			10,0
Errore Standard (SEM)			7,5
paired t-test			-3,463850733
df			9,0
p-value			0,007117445
